# Photobiomodulation versus stabilization splint, both combined with self-management, for acute temporomandibular myalgia: 48-week outcomes of a partially randomized controlled study

**DOI:** 10.1371/journal.pone.0360472

**Published:** 2026-10-27

**Authors:** Nguyen Ngoc Hoa, Toan Do, Viet Hai Hoang, To Thanh Dong, Tran Thị Minh Quyen, Tran Thai Binh

**Affiliations:** 1 Ha Noi Medical University, Hanoi, Vietnam; 2 108 Military Central Hospital, Hanoi, Vietnam; Massachusetts General Hospital, UNITED STATES OF AMERICA

## Abstract

**Background:**

Photobiomodulation (LLLT) is used for myogenous temporomandibular disorders (TMD), but longer-term comparative evidence versus stabilization splints alongside structured self-management remains limited. This study aims to compare pain reduction and maximum painless mouth opening after photobiomodulation+self-management (PBM + SM) versus stabilization splint+self-management (OS + SM), with an additional self-management–only comparator (SM), over 48 weeks.

**Methods:**

This partially randomized controlled study enrolled 66 patients with DC/TMD-diagnosed acute masticatory muscle pain. Forty-four participants consenting to intervention were randomized 1:1 to PBM + SM (660-nm diode laser, twice weekly, 10 sessions) or OS + SM (maxillary stabilization splint worn at night, 8 h/day for 2 months). Both intervention groups received the same SM programme (education, habit modification, and jaw exercises for 2 months). A non-randomized convenience group received SM alone (n = 22). Assessments were performed at baseline and weeks 2, 4, 12, 24, and 48. Primary outcomes were VAS pain (0–10) and maximum painless mouth opening (MPMO, mm). Secondary outcomes included masticatory muscle tenderness and Patient Global Impression of Change (PGIC). Longitudinal analyses used linear mixed-effects models.

**Results:**

All groups improved over time, with earlier improvement in both intervention arms. In the randomized comparison, PBM + SM and OS + SM showed comparable week-48 outcomes for VAS pain and MPMO. No significant between-group difference in masticatory muscle tender points was observed at week 48 after Holm adjustment. Comparisons involving the SM-only group were exploratory.

**Conclusions:**

Over 48 weeks, PBM + SM and OS + SM produced comparable improvements in pain and maximum painless mouth opening, with no significant between-group difference in masticatory muscle tender points at week 48.

**Trial Registration:**

ClinicalTrials.gov ID: NCT06419738

## Introduction

Temporomandibular disorders (TMD) comprise a heterogeneous group of musculoskeletal conditions affecting the temporomandibular joints, masticatory muscles, and associated structures, commonly presenting with pain, altered mandibular function, and joint sounds. TMD are also among the most common causes of nonodontogenic pain in the orofacial region [1,2]. The clinical manifestations of TMD significantly impair masticatory function and activities of daily living, leading to a diminished quality of life if left untreated or managed inappropriately [3]. Epidemiological data indicate that the community prevalence of TMD is steadily increasing, with estimated rates of approximately 31% in adults and 11% in adolescents [4]. Geographically, the prevalence in South America (47%) is notably higher than in Asia (33%) and Europe (29%) [5]. This rising prevalence entails an escalating disease burden and substantial healthcare expenditure; for instance, the total cost for TMD treatment in South Korea reached 721,447.5 USD in 2017 [6].

Given the considerable prevalence and functional burden of TMD, a consistent approach to assessment is important for both clinical care and research. The Diagnostic Criteria for Temporomandibular Disorders (DC/TMD) offer a structured approach for the clinical and research assessment of TMD. This framework addresses the disorder from two complementary perspectives: Axis I focuses on identifying the principal pain-related and intra-articular TMD diagnoses through standardized clinical assessment, whereas Axis II examines the psychosocial and behavioral context of the condition, including pain-related disability and limitations in jaw function [1].

Following diagnostic assessment, treatment is generally aimed at relieving pain, improving mandibular function, and reducing the impact of symptoms on everyday activities [7]. Among the diverse physiotherapy modalities available for TMD management, low-level laser therapy (LLLT) – also known as photobiomodulation (PBM) – has gained significant popularity due to its conservative nature and its analgesic, regenerative, and anti-inflammatory effects on target tissues [8]. While most literature supports its efficacy in pain reduction, its role in improving the range of mandibular motion remains a subject of ongoing controversy [9–11]. Current evidence suggests that PBM may offer clinical benefits across a range of conditions [12], however the certainty of these findings remains limited, and further well-designed trials with more consistent treatment protocols are needed before broader clinical implementation. In addition, PBM is better considered as an adjunct within a multimodal management approach rather than as a substitute for established standard therapies [13].

Recent trends in randomized controlled trials (RCTs) increasingly favor multimodal approaches, such as combining PBM with occlusal splints or pharmacological therapy [14–16]. Photobiomodulation is proposed as a potent adjunctive intervention for muscular TMD, facilitating pain relief and reducing inflammation to enhance mandibular function. Concurrently, self-management (SM) programs – comprising patient education, habit reversal, and jaw exercises – are recommended as first-line conservative treatments because they target modifiable behavioral factors [17,18]. The integration of PBM with SM may yield accelerated symptom relief and improve patient adherence to behavioral changes. In parallel, stabilization splints (OS) remain a standard mechanical approach to reduce functional overload, typically prescribed alongside SM programs [19].

Despite the widespread use of PBM and OS, direct comparative evidence on their efficacy as adjuncts to a standardized self-management program – particularly over the long term – is scarce [13,20]. Most existing trials report only short-term outcomes [21], yet TMD symptoms often fluctuate over time, and recurrence after conservative management has been reported, with recurrence rates varying by population and treatment modality [22,23]. Consequently, robust data extending beyond the immediate post-treatment phase is essential to validate treatment stability. To address this gap, the current study compares PBM + SM and OS + SM against a standalone SM group in patients with acute myogenous TMD over a 48-week follow-up period. It was hypothesized that PBM + SM and OS + SM would produce comparable improvements in VAS pain and maximum painless mouth opening, and that both combination therapies would be associated with more favourable 48-week outcomes than self-management alone.

## Materials and methods

### Study design

This study employed a partially randomized controlled design, comprising a two-arm randomized trial and an additional non-randomized self-management-only comparison group. The randomized trial compared photobiomodulation plus self-management (PBM + SM), registered on ClinicalTrials.gov as low-level laser therapy plus self-management (LLLT+SM), with occlusal splint plus self-management (OS + SM). Participants who consented to interventional treatment were randomized in a 1:1 ratio to receive either PBM + SM or OS + SM. Participants who declined interventional treatment were invited to receive self-management alone and were enrolled as a convenience comparison group until the planned sample size was reached (n = 22).

The trial was registered retrospectively because prospective registration had not been completed before participant recruitment began. At study initiation, the research team was not yet fully aware of the requirement for prospective registration of randomized clinical trials; however, the study protocol had received institutional ethics approval before participant enrolment and data collection. The authors confirm that all ongoing and related trials for this intervention are registered.

### Study setting

The study was conducted at the High-Tech Center for Odonto-Stomatology, School of Dentistry, Hanoi Medical University, Vietnam, from 2023-04-01 to 2025-04-01, with follow-up assessments at baseline and at weeks 2, 4, 12, 24, and 48.

### Participants

Participants were 16 or older, diagnosed with Temporomandibular Joint Disorders (TMD) according to the Diagnostic Criteria for TMD (DC/TMD). Before enrollment, participants were informed about the study procedures, potential benefits and risks, the voluntary nature of participation, the protection of personal information, and their right to discontinue participation at any time without any adverse consequences. Written informed consent was obtained from all participants prior to enrollment. For participants aged 16–17 years, written consent was also obtained from a parent or legal guardian. The study was conducted in accordance with the Declaration of Helsinki and received approval from Hanoi Medical University Institutional Ethical Review Board.

#### Inclusion criteria.

Acute myalgia of the temporalis and/or masseter muscles (pain duration <3 months) [24], maximum painless mouth opening <40 mm. All participants were required to demonstrate comprehension of the study protocol and be able to comply with examination procedures.

#### Exclusion criteria.

Patients with chronic TMD, characterized by persistent pain, oral edema, and discomfort preventing occlusal splint application, were excluded. Additionally, individuals with a history of severe neurological impairments, autoimmune joint or muscle diseases, advanced malignancies, psychiatric disorders, substance abuse, or recent neck/facial surgery or radiotherapy were ineligible. Concurrent treatments targeting the neck or temporomandibular joint within the past three months and the use of neuromuscular-affecting medications were exclusionary criteria. Pregnant women were also excluded from the study [25–27].

#### Group allocation and randomization.

Participants who consented to receive active intervention (n = 44) were assigned in a 1:1 ratio to PBM + SM or OS + SM using a computer-generated simple random sequence created in Microsoft Excel (no blocking). Allocation concealment was ensured by sequentially numbered, sealed opaque envelopes prepared by an independent staff member not involved in enrolment, treatment, or outcome assessment. After written informed consent and completion of baseline assessment, an investigator not involved in outcome assessment opened the next envelope in sequence and assigned participants to PBM + SM for even numbers and OS + SM for odd numbers.

### Interventions

#### Self-management programme (all groups).

All participants received a standardised self-management (SM) programme comprising education about TMD, behavioural modification (avoidance of parafunctional habits; sleep and head–neck–shoulder posture advice), dietary recommendations (soft diet; avoidance of hard foods), and jaw mobility exercises incorporating cold and warm compresses [2]. Participants were provided with written instructions and a demonstration video and were instructed to perform the exercises twice daily for 2 months (two sessions/day, three repetitions). Adherence support included twice-weekly clinic visits during the first 2 weeks, weekly reminder phone calls, completion of a daily monitoring form, and video-based feedback on exercise technique when required.

#### Photobiomodulation plus self-management (PBM + SM).

In addition to the self-management programme, participants received PBM using a diode laser (SiroLaser Blue, Dentsply Sirona) according to the previously described protocol [28]. The PBM treatment parameters are summarized in Table 1.

**Table 1:** Photobiomodulation treatment parameters and irradiation protocol.

Parameter	Treatment setting
Device	SiroLaser Blue, Dentsply Sirona
Light source	Diode laser
Wavelength	660 nm
Output power	25 mW
Energy density	2.5 J/cm²
Application mode	Contact mode
Probe orientation	Perpendicular to the target area
Irradiation sites	Predefined painful points over the temporalis (anterior, middle, posterior), masseter (upper, middle, lower), medial pterygoid insertion and five periarticular TMJ points (superior/anterior/lateral/posterior/posteroinferior to the condyle)
Irradiation time	20 s per site
Number of treatment sessions	10 sessions
Treatment frequency	Twice weekly
Treatment schedule	Nonconsecutive days

#### Occlusal splint plus self-management (OS + SM).

In addition to SM, participants received a maxillary stabilisation occlusal splint fabricated from autopolymerising acrylic resin using the Okeson technique [2]. The splint was adjusted to provide even occlusal contacts with canine guidance, and participants were instructed to wear it at night for approximately 8 hours/day for 2 months, with follow-up visits for fit and occlusal adjustment as needed.

### Outcomes

The primary outcomes were pain intensity and maximum painless mouth opening (MPMO). Pain intensity was assessed using a 10-cm unmarked visual analogue scale (VAS), anchored at the left end as “no pain” and at the right end as “unbearable pain”. Participants were instructed to draw a vertical mark on the line to indicate their pain intensity; the distance from 0 to the mark was then measured in centimetres using a caliper [29]. MPMO was measured as the interincisal distance (mm) between the incisal edges of the right maxillary and mandibular central incisors during maximal mouth opening without pain, using a millimetre ruler [30].

Secondary outcomes were the number of painful masticatory muscle points on palpation and patient global impression of change (PGIC). Masticatory muscle pain points were assessed following the standardised DC/TMD examination protocol. Bilateral palpation was performed over the temporalis (anterior/middle/posterior) and masseter (origin/body/insertion) using calibrated finger pressure (approximately 1 kg), applied perpendicular to the muscle for the standard duration; participants were asked to report pain in response to palpation, and the number of sites eliciting pain was summed as the total count of painful points [30]. PGIC was assessed at weeks 12, 24, and 48 using a 6-point global improvement scale (1 = much worse to 6 = symptom-free); for analysis, responses were grouped as symptom-free (6), improved (4–5), and not improved (1–3) [31].

Assessments were performed at baseline (T0) and at weeks 2 (T2), 4 (T4), 12 (T12), 24 (T24), and 48 (T48).

### Sample size calculation

Sample size was estimated a priori using G*Power 3.1 based on the primary outcome to ensure adequate power for the randomized comparison between the two active intervention arms (PBM + SM vs OS + SM). A conservative expected effect size was set at f = 0.444, derived from prior laser and occlusal splint studies, with α = 0.05 and 80% power [32]. The calculation yielded a required total sample size of 54 participants, corresponding to 18 participants per group for the planned three-group study. To account for potential attrition during the 48-week follow-up, the calculated sample size was increased by approximately 20% (54 × 1.20 = 64.8). To maintain equal group sizes, the target sample was rounded up to 66 participants, corresponding to 22 participants per group. Of these, participants in the two active intervention groups (PBM + SM and OS + SM) were randomized, whereas the SM-only group was recruited as a non-randomized convenience comparator and was matched in size to the intervention groups for comparability.

### Blinding

Due to the nature of the interventions (PBM and occlusal splint therapy), participants and the treating clinician could not be blinded. All treatment procedures were delivered by the principal investigator under the supervision of the study supervisors. Follow-up outcome assessments were conducted by an independent clinician experienced in TMD who remained blinded to treatment allocation. Participants were instructed not to disclose their treatment allocation during assessments. The statistician performed analyses using anonymised group codes.

### Patient and public involvement

No formal patient or public advisory group was convened. Informal feedback from patients during routine clinical consultations was used to refine participant information materials and clarify follow-up instructions.

### Statistical analysis

Statistical analyses were performed using Python 3.13.5 and statsmodels 0.14.6. Primary efficacy was assessed between the randomized groups (PBM + SM vs OS + SM) under the intention-to-treat principle. Repeated VAS and MPMO measurements at T0 and weeks 2, 4, 12, 24, and 48 were analysed using linear mixed-effects models with fixed effects for group, time (categorical), and group×time, and a participant-level random intercept. Tender-point counts were analysed using a Poisson generalized linear mixed-effects model. Overall group, time, and group×time effects were evaluated using omnibus tests, followed by model-based time-specific contrasts. Comparisons involving the non-randomized SM group were exploratory and adjusted for age, pain duration, baseline outcome, GAD-7, PHQ-9, and PHQ-15. Holm adjustment was applied to multiple time-specific contrasts within each outcome. Both unadjusted and Holm-adjusted p values were reported, with inference based on adjusted values. All tests were two-sided, with p < 0.05 considered statistically significant.

### Ethical approval & informed consent

This research has been approved according to certificate number 858/GCN-HĐĐĐ NCYSH-ĐHYHN issued by the Hanoi Medical University Institutional Ethical Review Board on March 14, 2023.

## Results

### Participant flow and baseline characteristics

A total of 120 participants were assessed for eligibility; 66 were enrolled and 54 were excluded. Twenty-two participants entered the SM group based on treatment preference after declining interventional therapy, while the remaining 44 were randomized equally to PBM + SM (n = 22) or OS + SM (n = 22). Follow-up was complete through week 12; retention at weeks 24 and 48 was 20/19 (SM), 20/19 (PBM + SM), and 22/21 (OS + SM). Participant recruitment and allocation are summarized in Fig 1.

**Fig 1:**
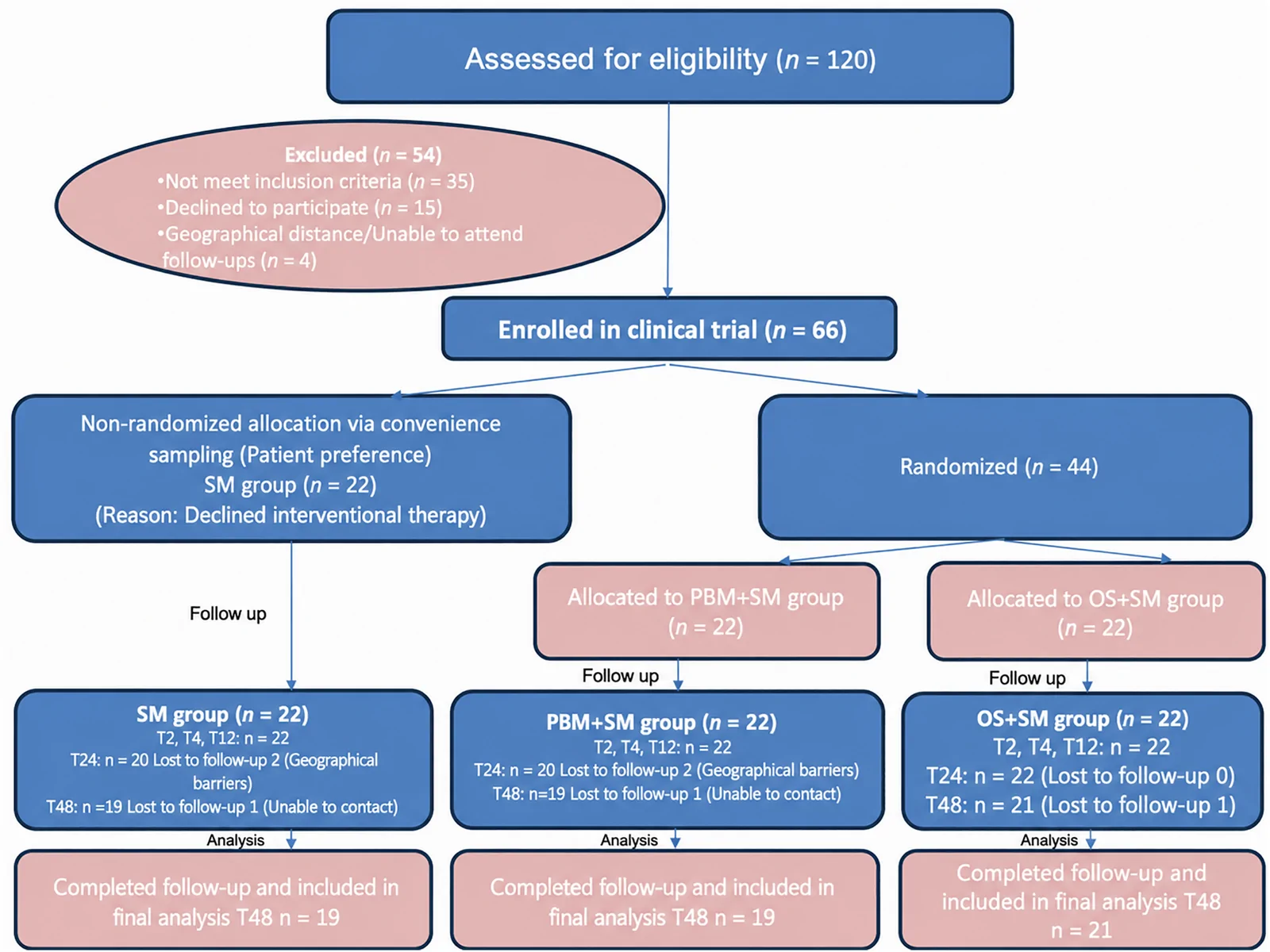
CONSORT flow diagram of participant recruitment, allocation, follow-up, and analysis.

Baseline demographic and clinical characteristics were generally comparable between the two randomized groups. Characteristics of the non-randomized SM group are also presented for descriptive comparison (Table 2).

**Table 2:** Baseline characteristics and key potential confounders.

Characteristic	SM Group n = 22	PBM + SM Group n = 22	OS + SM Group n = 22	Overall n = 66	p value
**Age (years)** **Median (IQR)**	28.0 (23.0–33.8)	31.0 (23.0–44.8)	28.0 (21.2–35.8)	29.0 (22.0–37.8)	.470
**Pain duration (days)** **Median (IQR)**	25.0 (10.2–37.5)	25.5 (11.0–56.2)	30.0 (16.2–60.0)	30.0 (11.0–60.0)	.520
**VAS T0** **(0–10), Mean ± SD**	5.5 ± 0.9	5.7 ± 1.3	6.1 ± 0.9	5.8 ± 1.1	.125
**GAD-7** **Median (IQR)**	3.0 (0.2–5.0)	4.5 (2.0–7.0)	4.0 (1.0–9.0)	4.0 (1.0–7.0)	.314
**PHQ-9** **Median (IQR)**	3.0 (0.2–5.8)	4.5 (1.2–6.8)	3.0 (1.2–8.8)	4.0 (1.0–6.0)	.556
**PHQ-15** **Median (IQR)**	3.5 (3.0–6.0)	5.5 (2.0–8.0)	4.5 (3.0–7.8)	5.0 (3.0–7.0)	.403

*Kruskal–Wallis Test.* Abbreviations: **SM** = Self-Management; **PBM** = Photobiomodulation; **OS** = Occlusal Splint; **VAS** = Visual Analog Scale; **GAD-7** = General Anxiety Disorder 7; **PHQ-9** = Patient Health Questionnaire 9; PHQ-15 = Patient Health Questionnaire 15. *Data are presented as mean (SD) for approximately normal variables and median (IQR) for non-normal variables (normality assessed by Shapiro–Wilk tests within each group). p-values were obtained using one-way ANOVA for approximately normal variables and Kruskal–Wallis tests for non-normal variables. Standardized mean differences were calculated to compare the randomized groups only (PBM + SM vs OS + SM) using means and SD. The SM group was a non-randomized convenience (exploratory) comparator, whereas PBM + SM and OS + SM were randomized.*

Sixty-six participants were included (22 per group). The median age was 29 years, and 62.1% were female (41/66). Baseline age, pain duration, VAS pain, and psychosocial scores (GAD-7, PHQ-9, PHQ-15) were comparable across groups (all p > 0.05). (Table 1)

#### Primary and secondary clinical outcomes.

Overall group, time, and group×time effects for the randomized comparison, followed by the week-48 model-based between-group contrasts, are summarized in Table 3.

**Table 3:** Overall model effects for the randomized comparison and model-based between-group contrasts at week 48.

Outcome	Model	Group effect, p		Time effect, p	Group × time interaction, p	
**Panel A. Overall fixed effects for the randomized comparison (PBM + SM vs OS + SM)**						
VAS (0–10)	LMM	.187		<.001	.520	
MPMO (mm)	LMM	.648		<.001	.938	
Masticatory muscle tender points	Poisson GLMM	.299		<.001	.040	
**Outcome**	**Comparison**	**Effect type**	**Effect estimate**	**95% CI**	**Unadjusted p**	**Holm-adjusted p**
**Panel B. Model-based between-group contrasts at week 48**						
VAS (0–10)	PBM vs OS	LMM (MD)	−0.31	−0.90 to 0.28	.301	.873
	PBM vs SM*	LMM (MD)	−1.31	−1.91 to −0.71	<.001	<.001
	OS vs SM*	LMM (MD)	−1.00	−1.59 to −0.41	<.001	.006
MPMO (mm)	PBM vs OS	LMM (MD)	−1.20	−4.36 to 1.95	.456	1.000
	PBM vs SM*	LMM (MD)	5.63	2.45 to 8.81	<.001	.006
	OS vs SM*	LMM (MD)	6.83	3.67 to 9.98	<.001	<.001
Masticatory muscle tender points	PBM vs OS	Poisson GLMM (RR)	0.10	0.01 to 0.72	.022	.157
	PBM vs SM*	Poisson GLMM (RR)	0.04	0.01 to 0.23	<.001	.005
	OS vs SM*	Poisson GLMM (RR)	0.37	0.18 to 0.77	.007	.066

*Note. Panel A presents omnibus tests for the randomized comparison (PBM + SM vs OS + SM). VAS and MPMO were analysed using linear mixed-effects models, and tender points using a Poisson GLMM, with a participant-level random intercept and all available observations under the intention-to-treat principle. Panel B presents week-48 model-based contrasts; Holm-adjusted* p *values account for multiple time-specific comparisons. Comparisons involving the non-randomized SM group were exploratory and adjusted for age, pre-treatment pain duration, baseline outcome, GAD-7, PHQ-9, and PHQ-15. CI, confidence interval; GLMM, generalized linear mixed-effects model; LMM, linear mixed-effects model; MD, mean difference; MPMO, maximum painless mouth opening; OS, occlusal splint; PBM, photobiomodulation; RR, rate ratio; SM, self-management; VAS, visual analogue scale.*

In the randomized comparison, significant time effects were observed for VAS, MPMO, and masticatory muscle tender points (all p < .001). No significant overall group effects or group×time interactions were found for VAS or MPMO, whereas tender points showed a significant group×time interaction (p = .040). At week 48, PBM + SM and OS + SM did not differ significantly in VAS, MPMO, or tender points after Holm adjustment. Exploratory comparisons with the non-randomized SM group showed more favourable VAS and MPMO outcomes for both active treatments; PBM + SM also showed fewer tender points than SM, whereas the OS + SM versus SM comparison was no longer significant after Holm adjustment (Table 2).

VAS scores decreased over time in all three groups, with greater reductions observed in the two active treatment groups, as illustrated in Fig 2.

**Fig 2:**
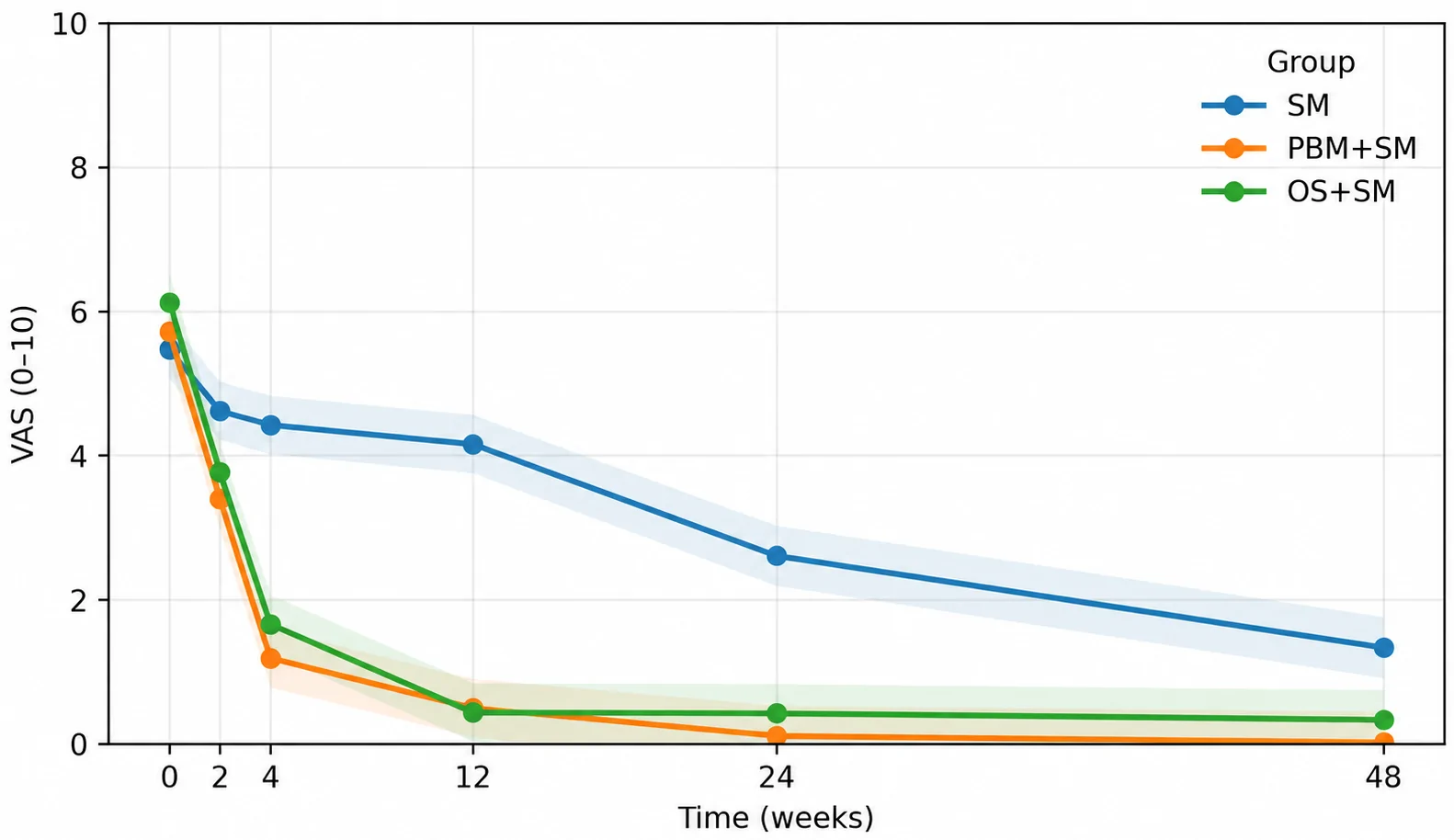
VAS over time (T0–T48). *Points show model-based estimated marginal means, and shaded bands indicate 95% confidence intervals from a linear mixed-effects model including fixed effects for group, time, and their interaction, with a random intercept for participant*.

Observed mean VAS values were comparable at baseline (T0: SM 5.47, PBM + SM 5.73, OS + SM 6.12) and then declined sharply by week 2 in the interventional groups (T2: 3.40 in PBM + SM and 3.77 in OS + SM), whereas the SM group showed a smaller change (T2: 4.62). By week 4, VAS was near 1 in PBM + SM (1.19) and OS + SM (1.66) but remained higher in SM (4.42), and this separation persisted through week 48 (T48: SM 1.43, PBM + SM 0.09, OS + SM 0.28) (Table S2 in S1 File).

In model-based contrasts, PBM + SM and OS + SM did not differ from each other at any follow-up time point (T48: MD −0.31, 95% CI −0.90 to 0.28; p = .301). Both interventional groups showed consistently lower VAS than SM from week 2 through week 48 (PBM + SM vs SM: MD −1.22 at T2 and MD −1.31 at T48; both p < .001 with Holm/FDR adjustment; OS + SM vs SM: MD −0.85 at T2 and MD −1.00 at T48; adjusted p-values remained significant). Comparisons involving SM should be interpreted as exploratory (nonrandomized). (Table S3 in S1 File).

MPMO increased over time in all groups (Fig 3). The two randomized intervention groups (PBM + SM and OS + SM) showed larger and earlier increases than the non-randomized SM comparator, with descriptive separation apparent from week 4 and maintained through week 48.

**Fig 3:**
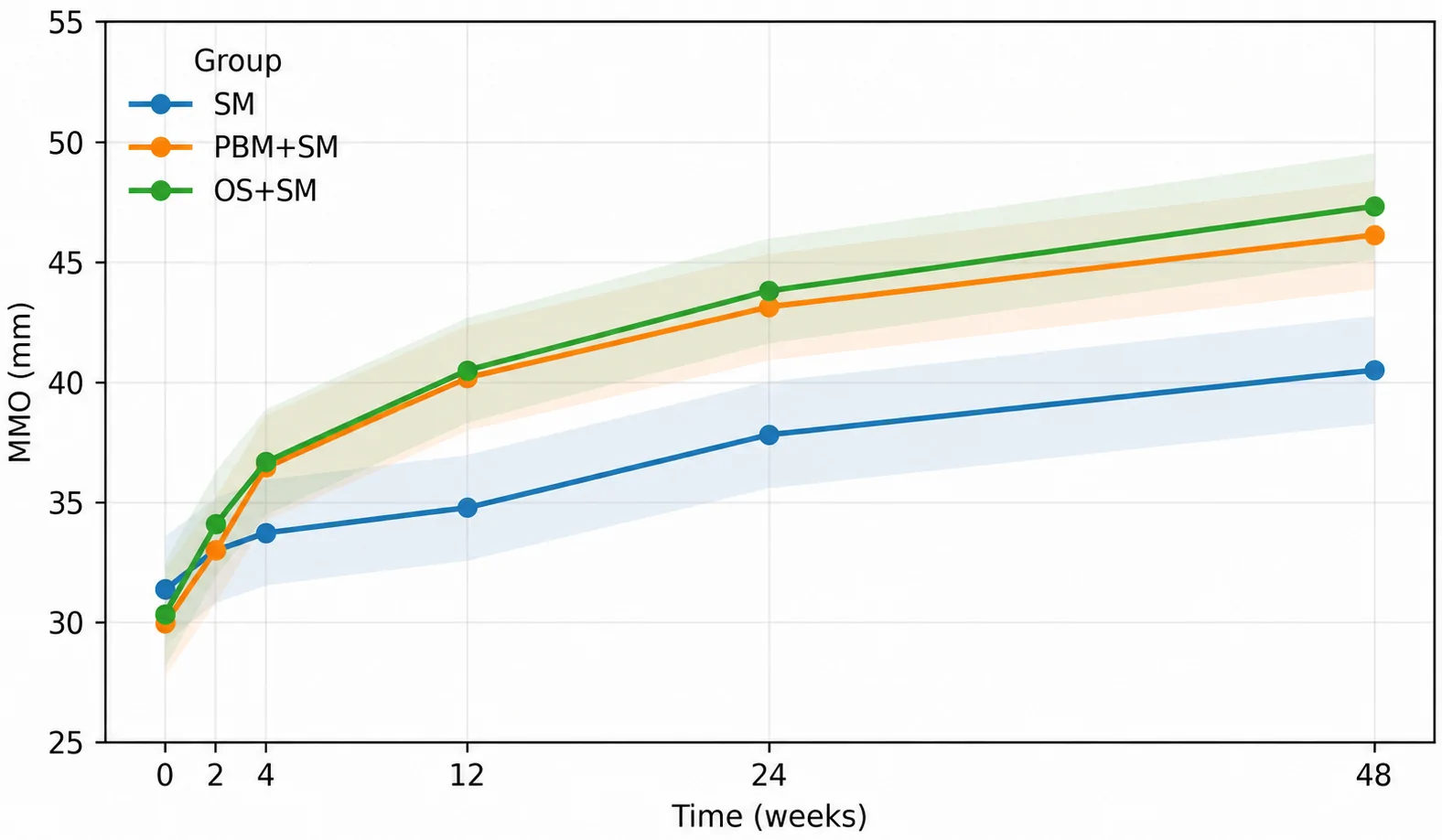
Maximum painless mouth opening (MPMO) over time (T0–T48). *Points show model-based estimated marginal means; shaded bands indicate 95% confidence intervals from a linear mixed-effects model including fixed effects for group, time, and the group×time interaction, with a participant-level random intercept. The SM group was a non-randomized convenience comparator*.

In the observed data (Table S2 in S1 File), mean MPMO increased from 31.36 to 40.63 mm in the SM group, from 29.95 to 46.16 mm in the PBM + SM group, and from 30.32 to 47.33 mm in the OS + SM group (T0–T48). Between-group differences were apparent at week 4 (SM 33.73 mm vs 36.45 mm and 36.68 mm) and persisted at week 48 (SM 40.63 mm vs 46.16 mm and 47.33 mm).

#### Patient global impression of change.

Fig 4 and Table S4 in S1 File demonstrate a trend toward superior Patient Global Impression of Change (PGIC) outcomes in the two active intervention groups throughout the follow-up period.

**Fig 4:**
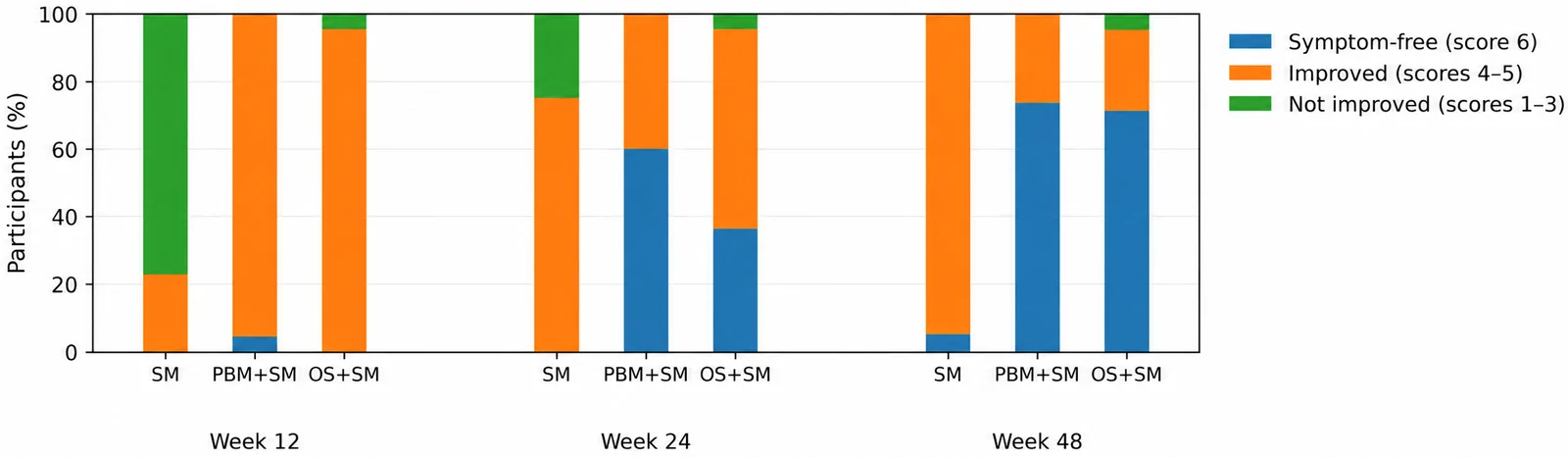
Patient Global Impression of Change (PGIC) distribution at weeks 12, 24, and 48 by treatment group.

At week 12, the majority of patients in both the PBM + SM and OS + SM groups were “improved” (95.5%), whereas the “not improved” category predominated in the SM group (25%). By week 24, both active intervention groups showed a distinct shift toward an “symptom-free” status – a trend that was maintained through week 48, with a concomitantly very low rate of “no improvement.” Furthermore, the distribution of PGIC scores indicated that the PBM + SM and OS + SM groups reached higher evaluative ratings (5–6) at an earlier stage.

## Discussion

The current study compared the integration of PBM + SM and OS + SM against standalone self-management (SM) for acute TMD-related masticatory muscle pain. Utilizing a mixed-effects model for longitudinal analysis, our 48-week follow-up showed that all groups improved over time, but the active intervention arms achieved clinically meaningful recovery earlier. A key finding is that PBM + SM and OS + SM demonstrated comparable long-term efficacy in reducing VAS pain and increasing maximum painless mouth opening (MPMO). This suggests that PBM may offer a more effective targeted resolution for localized muscle sensitivity over a prolonged period.

### Variations in pain intensity (VAS scores)

A clinically relevant observation is the different *rate* of pain reduction across groups. Although baseline pain levels were comparable, VAS decreased earlier in both active intervention groups than in SM alone, and this separation emerged from early follow-up and was maintained through 48 weeks. In practice, earlier symptom relief is important because it may facilitate participation in home exercises and behavioural modification, thereby reinforcing adherence and supporting sustained improvement.

Prior trials and reviews have generally supported that PBM can reduce pain in myogenous TMD, although reported magnitudes vary across protocols [10,33,34]. In comparative studies, VAS after 3–6 months commonly stabilizes at low-to-moderate levels, and rapid reductions immediately after completing laser sessions have also been described [34,35]. When combined with a standardized self-management programme, PBM and occlusal splint therapy were associated with earlier pain improvement and sustained favourable outcomes over the longer follow-up period. Importantly, in the randomized comparison, no significant difference between PBM + SM and OS + SM in VAS was found at week 48, consistent with several studies reporting broadly similar pain reduction between laser and stabilization splint approaches [33,36].

Most published trials have follow-up shorter than 24 weeks and often evaluate single modalities rather than combination strategies [11,35]. These limitations limit inferences about durability and about how active interventions perform when delivered alongside behavioural and exercise-based care. In the present study, both active arms reached near-minimal pain levels by mid-follow-up, which is not consistently reported in earlier literature. We interpret this as compatible with a synergistic effect of combining an adjunctive active modality with structured education, habit modification, and home-based exercises [32,37,38]. The early response in the PBM + SM arm is clinically noteworthy. The largest pain reduction occurred within the first 2–4 weeks, corresponding to approximately four laser sessions, suggesting that laser may offer early analgesia that can support function and improve tolerance for training and behavioural change. Similar early improvements have been reported in trials evaluating PBM or laser-related approaches across short intervals [32,37,38]. In addition, recent comparative PBM studies in myogenous TMD have reinforced clinically meaningful pain reductions and have explored PBM as monotherapy and as an adjunct to splints [15].

In contrast, previous evidence suggested that laser-based PBM was less effective than occlusal splint therapy [39]. The splint type used in that study was an anterior repositioning splint, unlike the stabilization splints utilized in most previous studies, and the author employed the Verbal Numeric Rating Scale (VNRS) for pain assessment. Some authors have concluded that PBM reduces pain in the masseter muscle but is ineffective for the temporalis muscle [40] and does not improve mandibular function [11]. RCTs comparing active PBM with inactive “sham” laser groups suggested that laser groups did not show superior pain improvement over placebos [28]. It has been reported that significant reductions in pain intensity occurred in both the laser and placebo groups, suggesting that symptom improvement may be largely attributable to the placebo effect or the natural fluctuating course of TMD [32]. This raises the hypothesis that the observed efficacy of laser therapy could be a placebo effect or a coincidence with the disorder's natural remission phase, rather than a direct effect of PBM. Our study partially addresses this concern by using a standalone SM group as a control. While the SM group also showed symptom improvement, the magnitude and rate of pain reduction were significantly lower than those in the PBM and OS combination groups, and the SM group required more time to achieve comparable results.

### Variations in masticatory muscle tender points

Tender point outcomes add clinically relevant nuance beyond global pain intensity scores. At week 48, the unadjusted analysis suggested fewer masticatory muscle tender points with PBM + SM than with OS + SM; however, this difference did not remain statistically significant after Holm adjustment. In exploratory comparisons with the non-randomized SM group, PBM + SM remained associated with fewer tender points after adjustment, whereas the OS + SM versus SM comparison no longer reached statistical significance. These findings suggest a potential benefit of PBM in reducing localized muscle tenderness, but the evidence is not sufficient to establish superiority over occlusal splint therapy. The results should therefore be interpreted cautiously, particularly given the small sample size and the exploratory nature of comparisons involving the SM group. Previous studies have reported reductions in muscle tenderness following both PBM and occlusal splint therapy [33,41–43]. However, the literature remains mixed, particularly for pressure pain threshold outcomes, with some studies reporting improvement while others show no meaningful change [11,44,45]. Such inconsistency likely results from heterogeneity in assessment methods and examined sites. The current study used manual palpation with standardized pressure following DC/TMD procedures (1 kg/cm^2^ for masticatory muscles and 0.5 kg/cm^2^ for TMJ/surrounding areas. Standardized palpation is clinically pragmatic and aligned with DC/TMD, whereas algometry can provide an objective threshold measure but is sensitive to calibration and technique. Previous studies have differed in whether cervical or shoulder muscles were included in the assessment [33,41]; in this study, examination was restricted to the primary masticatory muscles, particularly the masseter and temporalis, which are commonly involved in myogenous TMD. Longitudinal data also suggested that differences between PBM + SM and OS + SM in tender-point counts were less apparent during the early follow-up and showed a different pattern over time. This may indicate that changes in muscle sensitivity evolve gradually, although the between-group difference was not definitive after adjustment for multiple comparisons.

### Improvement in maximum painless mouth opening (MPMO)

MPMO improved progressively over time in all groups, with earlier improvement in the two active intervention arms. At week 48, both PBM + SM and OS + SM achieved significantly greater MPMO than SM, whereas no significant difference was observed between the two randomized arms. Several comparative studies have reported similar functional improvements in both laser and splint groups, particularly in the early treatment period [33,35]. In addition, evidence suggests that combining exercises with laser may enhance functional recovery compared with laser alone, supporting a synergistic mechanism between pain relief and active rehabilitation [46].

However, the effect of PBM on mouth opening remains controversial. Some studies and reviews have concluded that laser improves pain but does not consistently translate into improved mandibular function or maximum opening [11,13,47]. A previous study reported a modest improvement in vertical mouth opening following PBM, with increases of approximately 10–20% [10]. These discrepant findings may be explained by differences in baseline functional limitation, chronicity, and protocol heterogeneity (wavelength, energy density, number of sessions, frequency, and irradiation sites). Trials delivering a limited number of sessions or using less standardized control conditions may be less likely to detect functional benefits. Prior research indicates that a greater number of PBM sessions may be associated with greater pain reduction, improved maximum painless mouth opening, and better quality of life [48].

### PGIC and patient-perceived improvement

The Patient Global Impression of Change (PGIC) provided a complementary patient-reported perspective alongside clinician-assessed and performance-based outcomes. Patients in the active intervention arms shifted earlier toward “improved” categories and showed progressive movement toward “symptom-free” at later follow-up, whereas SM alone showed slower and less complete recovery. This patient-perceived trajectory mirrors the objective trends in pain and function, supporting the clinical relevance of adding an active modality to a structured SM foundation. Patient-reported global improvement after PBM has been reported previously [49], and splint trials have similarly shown meaningful proportions of patients reporting improvement at short-term follow-up [31].

### Clinical practicality, strengths, and limitations

From a clinical perspective, PBM requires equipment and repeated visits but involves a relatively standardized technique; splint therapy requires fabrication and adjustment but can be maintained with fewer in-clinic sessions once delivered. Clinically, practitioners should provide comprehensive counseling and choose individualized treatment plans for each patient. Key strengths include the long follow-up (48 weeks), longitudinal modelling using mixed-effects methods, standardized DC/TMD-based examination procedures, and adjustment for multiplicity to reduce type I error from repeated comparisons. The study also assessed both patient-reported and clinician-examined outcomes, providing convergent clinical evidence.

Limitations include the non-randomized, preference-based SM comparator, which renders comparisons involving the SM group exploratory and potentially vulnerable to residual confounding; the inability to blind participants and treating clinicians, although outcome assessors were blinded; and the relatively small sample size with some late attrition, although dropout did not differ meaningfully between the randomized arms. The modest sample size may also limit the precision of the estimates; therefore, p values, particularly those from secondary and exploratory comparisons, should be interpreted cautiously together with the corresponding effect estimates and 95% confidence intervals. Given the ongoing heterogeneity in PBM parameters, larger trials with standardized dosimetry and longer post-treatment follow-up are warranted to confirm the durability and generalizability of these findings.

## Conclusions

Over 48 weeks, PBM + SM and OS + SM showed comparable improvements in VAS pain and maximum painless mouth opening, with no statistically significant differences between the randomized groups. Exploratory comparisons with the non-randomized SM group suggested more favourable 48-week pain and mouth-opening outcomes with both combination therapies and should be interpreted cautiously. These findings provide clinically relevant longer-term evidence supporting the use of PBM or occlusal splint therapy alongside structured self-management for acute myogenous TMD.

## Supporting information

S1 File Supplementary Tables S1–S4. (DOCX)

S2 Data De-identified raw dataset underlying the analyses. (XLSX)

S3 Protocol. Original study protocol in Vietnamese.
(PDF)

S4 Protocol. English translation of the original study protocol.
(PDF)
