# Supplementary material for: Photobiomodulation versus stabilization splint, both combined with self-management, for acute temporomandibular myalgia: 48-week outcomes of a partially randomized controlled study
Source: PLoS One. 2026 Oct 27;21(10):e0360472. doi: 10.1371/journal.pone.0360472 (PMC13712594; doi:10.1371/journal.pone.0360472)
Supplement: Supplementary file 1 [file pone.0360472.s003.pdf]

TRƯỜNG ĐẠI HỌC Y HÀ NỘI
HỘI ĐỒNG ĐẠO ĐỨC TRONG NGHIÊN CỨU Y SINH HỌC

ĐỀ CƯƠNG NGHIÊN CỨU

ĐẶC ĐIỂM LÂM SÀNG, TÂM LÝ XÃ HỘI, HÀNH VI VÀ HIỆU QUẢ ĐIỀU TRỊ CỦA LASER NĂNG LƯỢNG THẤP TRÊN BỆNH NHÂN RỐI LOẠN KHỚP THÁI DƯƠNG HÀM

Nghiên cứu viên chính: Nguyễn Ngọc Hoa

Đơn vị công tác: Trường Đại học Y Hà Nội

Phiên bản đề cương: 1.0

Ngày nộp đề cương: 12/01/2023

Thời gian thực hiện nghiên cứu: 2023–2025

NGHIÊN CỨU VIÊN CHÍNH
(Ký và ghi rõ họ tên)

Nguyễn Ngọc Hoa

Hà Nội, 2023

I. THÔNG TIN CHUNG VỀ ĐỀ TÀI

1. Tình trạng hồ sơ: Nộp mới
2. Hình thức xét duyệt: Xét duyệt lần đầu
3. Tên đề tài: Đặc điểm lâm sàng, tâm lý xã hội, hành vi và hiệu quả điều trị của laser năng lượng thấp trên bệnh nhân rối loạn thái dương hàm
4. Mã đề tài: NCS
5. Mã số hồ sơ: 858
6. Thông tin nghiên cứu viên chính:
   Họ và tên: Nguyễn Ngọc Hoa
   Giới tính: Nữ
   Ngày sinh:
   Chức vụ: Giảng viên
   Đơn vị công tác: Trường Đại học Y Hà Nội
   Học hàm: Không
   Học vị: Bác sỹ nội trú
   Điện thoại:
   Email: nguyenngochoa@hmu.edu.vn
7. Đơn vị chủ trì: Trường Đại học Y Hà Nội

7.1 Địa chỉ: Số 1 Tôn Thất Tùng, Đống Đa, Hà Nội
7.2 Người đại diện: Nguyễn Hữu Tú
7.3 Chức vụ: Hiệu trưởng
7.4 Số điện thoại:
7.5 Email: nguyenhuutu@hmu.edu.vn

8. Đơn vị tài trợ: Không
9. Tổng kinh phí thực hiện đề tài: 135.700.000 VNĐ
10. Thời gian bắt đầu: tháng 4/2023 Thời gian kết thúc: tháng 10/2025
11. Phân loại đề tài: đề tài của Nghiên cứu sinh
12. Thư ký đề tài:
    - Tên tài khoản: nguyenngochoa@hmu.edu.vn
    - Học và tên: Nguyễn Ngọc Hoa
    - Học hàm: Không
    - Học vị: Bác sỹ nội trú
    - Đơn vị công tác: Trường Đại học Y Hà Nội
    - Email: nguyenngochoa@hmu.edu.vn
    - Số điện thoại:
13. Lịch sử xét duyệt bởi Hội đồng đạo đức:

Lần xét duyệt	Kết quả	Số chấp thuận	Ngày chấp thuận	Phiên bản hồ sơ
1	Thông qua	858	14/3/2026	1

14. Đơn xin xét duyệt: có đính kèm

II. THÔNG TIN CHỨNG NHẬN CHẤP THUẬN ĐẠO ĐỨC

Bằng tiếng Việt:

- Tên đề tài: Đặc điểm lâm sàng, tâm lý xã hội, hành vi và hiệu quả điều trị của laser năng lượng thấp trên bệnh nhân rối loạn thái dương hàm
- Tên nghiên cứu viên chính: Nguyễn Ngọc Hoa
- Đơn vị tài trợ: Không
- Cơ quan chủ trì: Trường Đại học Y Hà Nội

- Địa điểm triển khai: các cơ sở thực hành của Viện đào tạo Răng Hàm Mặt - Trường Đại học Y Hà Nội: Trung tâm kỹ thuật cao Răng Hàm Mặt, Trung tâm nha khoa 225 Trường Chinh, Khoa Răng Hàm Mặt – Bệnh viện Đại học Y Hà Nội.
- Thời gian bắt đầu: 1/4/2023 Thời gian kết thúc: 31/10/2025
- Danh sách tài liệu được Hội đồng xem xét và thông qua:

STT	Tên tài liệu	Phiên bản số	Ngày/tháng/năm
1	Đề cương chi tiết	01	20/01/2023
2	Bệnh án nghiên cứu	01	20/01/2023
3	Giấy chấp thuận tham gia nghiên cứu cắt ngang	01	20/01/2023
4	Giấy chấp thuận tham gia nghiên cứu thử nghiệm lâm sàng	01	20/01/2023

ĐỀ CƯƠNG NGHIÊN CỨU

Tên đề tài: Đặc điểm lâm sàng, tâm lý xã hội, hành vi và hiệu quả điều trị của laser năng lượng thấp trên bệnh nhân rối loạn khớp thái dương hàm

Đặt vấn đề:

Rối loạn khớp thái dương hàm (Temporomandibular Disorders - TMDs) được coi là một tập hợp các bệnh lý cơ xương liên quan giữa các cơ nhai, khớp thái dương hàm và các cấu trúc liên quan. Đây là nguyên nhân chính gây đau có nguồn gốc không do răng trong vùng đầu mặt.

Các triệu chứng của rối loạn khớp thái dương hàm ảnh hưởng nghiêm trọng đến chức năng, hoạt động hàng ngày của bệnh nhân, làm giảm chất lượng cuộc sống nếu không được điều trị hoặc điều trị không đúng. Các nghiên cứu dịch tễ học chỉ ra rằng khoảng 10% –15% dân số nói chung mắc rối loạn khớp thái dương hàm. Trong đó 5% số người được hỏi có nhu cầu điều trị. Tại Việt Nam, tỷ lệ bệnh nhân có triệu chứng rối loạn khớp thái dương hàm dao động từ 20,6 % đến 60,5 % tuỳ nghiên cứu. Từ những nghiên cứu trên cho thấy, tỷ lệ mắc bệnh và triệu chứng có thể khác nhau trong các quần thể nhưng nhu cầu điều trị rối loạn khớp thái dương hàm trên thế giới và nhất là ở Việt Nam rất lớn. Do căn nguyên của rối loạn khớp thái dương hàm là đa yếu tố nên phương pháp điều trị sẽ rộng và đa dạng. Rất nhiều nghiên cứu gần đây khuyến nghị các liệu pháp điều trị bảo tồn, không xâm lấn, có thể đảo ngược như: liệu pháp dược lý, các liệu pháp không dùng thuốc bao gồm máng nhai, kim khô, vật lý trị liệu và phục hồi chức năng, liệu pháp siêu âm, kích thích dây thần kinh điện qua da, kỹ thuật thư giãn, châm cứu, tập thể dục kéo giãn, liệu pháp xoa bóp, liệu pháp laser năng lượng thấp và điều trị

tâm lý. Laser năng lượng thấp đã được chứng minh là an toàn, hầu như không có tác dụng phụ, chi phí điều trị vừa phải, số lần điều trị không nhiều, dễ áp dụng. Tại Việt Nam, hiện chưa có nghiên cứu nào về hiệu quả điều trị của laser năng lượng thấp và việc áp dụng kỹ thuật này vẫn đang hạn chế tại một số ít trung tâm lớn. Việc phổ biến rộng rãi kỹ thuật này sẽ đem lại lợi ích điều trị to lớn cho bệnh nhân, giúp giảm đau, tăng mức độ há miệng và chuyển động của hàm dưới. Trong nghiên cứu này, chúng tôi tiến hành nghiên cứu đặc điểm lâm sàng, tâm lý xã hội, hành vi của 120 bệnh nhân rối loạn khớp thái dương hàm đến khám tại ba cơ sở thực hành của Viện đào tạo Răng Hàm Mặt, Trường Đại học Y Hà Nội. Chúng tôi sẽ lựa chọn 66 bệnh nhân tại Trung tâm Kỹ thuật cao Răng Hàm Mặt, Trường Đại học Y Hà Nội tham gia tiếp thử nghiệm lâm sàng để đánh giá hiệu quả điều trị của laser năng lượng thấp trong điều trị rối loạn khớp thái dương hàm.

Mục tiêu nghiên cứu:

1. *Mô tả đặc điểm lâm sàng của bệnh nhân rối loạn khớp thái dương hàm tại một số cơ sở thực hành của Viện đào tạo Răng Hàm Mặt, trường Đại học Y Hà Nội.*
2. *Phân tích một số yếu tố tâm lý xã hội, hành vi của bệnh nhân rối loạn khớp thái dương hàm.*
3. *Đánh giá kết quả của laser năng lượng thấp trong điều trị rối loạn khớp thái dương hàm.*

Đối tượng nghiên cứu: Nghiên cứu của chúng tôi gồm 2 phần: nghiên cứu mô tả cắt ngang và thử nghiệm lâm sàng có đối chứng

Trong nghiên cứu mô tả cắt ngang:

Tiêu chuẩn lựa chọn: những bệnh nhân đến khám, được chẩn đoán rối loạn khớp thái dương hàm tại ba cơ sở thực hành của Viện đào tạo Răng Hàm Mặt, Trường Đại học Y Hà Nội: Trung tâm Kỹ thuật cao RHM, Trung tâm Nha khoa 225 Trường Chinh, Khoa RHM - Bệnh viện Đại học Y Hà Nội trong thời gian từ tháng 4/2023 đến tháng 10/2025. Tất cả các bệnh nhân từ 16 tuổi trở lên, có khả năng lĩnh hội ý kiến của bác sỹ và tuân thủ quy trình khám.

Tiêu chuẩn loại trừ:

- Những bệnh nhân có tiền sử rối loạn thần kinh nghiêm trọng, bệnh tự miễn dịch khớp và cơ, ung thư ác tính tiến triển, tiền sử bệnh tâm thần, tiền sử nghiện rượu, lạm dụng ma túy và thuốc giảm đau, bệnh nhân trải qua phẫu thuật và/ hoặc xạ trị trên vùng cổ và mặt, các phương pháp điều trị khác ở vùng cổ và khớp thái dương hàm trong vòng 3 tháng, sử dụng thuốc có thể ảnh hưởng đến hệ thần kinh cơ
- Bệnh nhân mang thai

Trong nghiên cứu thử nghiệm lâm sàng có đối chứng:

Bệnh nhân được tuyển chọn song song từ nghiên cứu mô tả cắt ngang tại Trung tâm Kỹ thuật cao khám chữa bệnh RHM, đồng ý tham gia nghiên cứu.

Tiêu chuẩn lựa chọn: BN được chẩn đoán đau cơ thái dương và/ hoặc cơ cắn cấp tính (đau dưới 3 tháng), biên độ há miệng tối đa không đau dưới 40 mm

Tiêu chuẩn loại trừ: BN đau mạn tính (đau từ 3 tháng trở lên), thoái hoá khớp, sưng đau trong miệng cản trở việc đeo máng nhai.

Thời gian và địa điểm nghiên cứu: từ tháng 4/2023 đến tháng 10/2025 tại Trung tâm kỹ thuật cao Răng Hàm Mặt, Trường Đại học Y Hà Nội.

Phương pháp nghiên cứu:

Nghiên cứu mô tả cắt ngang: cỡ mẫu 120 bệnh nhân

Thử nghiệm lâm sàng có đối chứng: cỡ mẫu 66 bệnh nhân

Quy trình nghiên cứu:

Đối với nghiên cứu mô tả cắt ngang:

- *Phỏng vấn: Khi bệnh nhân đến khám rối loạn khớp thái dương hàm, bệnh nhân sẽ được hỏi các thông tin hành chính: họ tên, tuổi, giới, địa chỉ, điện thoại, lý do đến khám. Bệnh nhân sẽ được phát phiếu thu thập thông tin và được bác sỹ hướng dẫn trả lời. Các phiếu bao gồm: phiếu câu hỏi triệu chứng, phiếu phỏng vấn đau theo thang điểm VAS, phiếu đánh giá thang điểm đau mạn tính, phiếu đánh giá hạn chế chức năng hàm dưới, phiếu đánh giá tình trạng trầm cảm, phiếu đánh giá tình trạng lo lắng, phiếu câu hỏi các thói quen miệng, phiếu đánh giá chất lượng cuộc sống. Bệnh nhân sẽ trả lời các phiếu câu hỏi này trong buổi khám đầu tiên.*
- *Bác sỹ sẽ tiến hành khám khớp thái dương hàm và các cấu trúc liên quan cho bệnh nhân theo đúng quy trình chuẩn. Bệnh nhân cần phối hợp với bác sỹ trong quá trình khám để đạt được kết quả tốt nhất.*
- *Bác sỹ sẽ chỉ định chụp MRI hoặc CT Cone Beam tuỳ tình trạng bệnh lý của bệnh nhân để đưa ra chẩn đoán xác định.*
- *Toàn bộ quy trình phỏng vấn, khám và các lần khám được thực hiện bởi tác giả, dưới sự giám sát của thầy hướng dẫn.*

Đối với thử nghiệm lâm sàng có đối chứng:

Bệnh nhân phù hợp với tiêu chuẩn chọn mẫu sẽ được tư vấn điều trị. Trong số các bệnh nhân không chấp nhận điều trị vì điều kiện kinh tế, chúng tôi thuyết phục tham gia nhóm chứng (nhóm 1). Nhóm 1 gồm 22 bệnh nhân sẽ được điều chỉnh hành vi và được lấy lần lượt theo thứ tự đến khám cho đến khi đủ số lượng. 44 bệnh nhân đồng ý điều trị sẽ được ngẫu nhiên hoá vào 2 nhóm còn lại bằng cách sử dụng phần mềm Excel để tạo bảng số ngẫu nhiên. Sau đó cho

vào phong bì, dán kín và đánh số thứ tự liên tục ở ba nơi nghiên cứu (khâu này được thực hiện bởi người không tham gia trực tiếp vào nhóm nghiên cứu).

Sau khi bệnh nhân đạt đủ tiêu chuẩn (tiêu chuẩn lựa chọn, loại trừ) và ký giấy đồng ý tham gia nghiên cứu, bác sỹ nhóm nghiên cứu sẽ mở bao thư theo đúng số liên tiếp quy định sẵn và phân đối tượng vào nhóm theo nguyên tắc: chọn số chẵn vào nhóm 2, số còn lại vào nhóm 3.

+ Nhóm 2: Điều trị laser năng lượng thấp, giáo dục bệnh nhân, điều chỉnh hành vi

+ Nhóm 3: Điều trị bằng máng ổn định, giáo dục bệnh nhân, điều chỉnh hành vi.

Bệnh nhân được hướng dẫn thực hiện và tuân thủ chặt chẽ các bài tập vận động, tự trị liệu tại nhà, điều chỉnh hành vi nhằm thay đổi các thói quen không tốt như: tư thế đầu, cổ, vai, những vấn đề liên quan đến giấc ngủ, ăn uống.

Giả thuyết nghiên cứu được chúng tôi đưa ra là: ‘‘Trên bệnh nhân rối loạn khớp thái dương hàm, hai phương pháp điều trị phối hợp gồm laser năng lượng thấp kết hợp điều chỉnh hành vi và máng nhai kết hợp điều chỉnh hành vi đều cải thiện các kết quả điều trị theo thời gian, bao gồm các chỉ số lâm sàng, chức năng và cảm nhận của người bệnh. Nghiên cứu tập trung so sánh hiệu quả giữa hai phương pháp này trong nhóm bệnh nhân được phân bố ngẫu nhiên. Ngoài ra, kết quả của hai chiến lược điều trị phối hợp cũng được đối chiếu bổ sung với nhóm điều chỉnh hành vi đơn thuần.’’

Sau khi được điều trị, bệnh nhân sẽ được hẹn tái khám sau 2 tuần, 4 tuần, 12 tuần, 24 tuần, 48 tuần để:

- Phỏng vấn: phiếu phỏng vấn đau theo thang điểm VAS, phiếu đánh giá hạn chế chức năng hàm dưới JFLS-8, đánh giá mức cải thiện sau điều trị.

- Khám xác định: số điểm đau cơ, biên độ há miệng tối đa không đau, tiếng kêu khớp, biên độ chuyển động sang bên, ra trước.

QUẢN LÝ, PHÂN TÍCH SỐ LIỆU

Các kết cục được đánh giá giữa các nhóm ngẫu nhiên (LLLT+SM so với OS+SM) theo nguyên tắc phân tích *intention-to-treat*. Các phép đo lường lặp lại tại thời điểm T0 và các tuần 2, 4, 12, 24 và 48 được phân tích bằng mô hình hiệu ứng hỗn hợp tuyến tính (*linear mixed-effects models*). Giá trị $p < 0,05$ được coi là có ý nghĩa thống kê.

Dự kiến kết quả nghiên cứu:

(1) *Mô tả đặc điểm lâm sàng của bệnh nhân rối loạn khớp thái dương hàm tại một số cơ sở thực hành của Viện đào tạo Răng Hàm Mặt, trường Đại học Y Hà Nội. (2) Phân tích một số yếu tố tâm lý xã hội, hành vi của bệnh nhân rối loạn khớp thái dương hàm. (3) Đánh giá kết quả của laser năng lượng thấp trong điều trị rối loạn khớp thái dương hàm.*

III. THÔNG TIN SẢN PHẨM THỬ NGHIỆM

(Thuốc, trang thiết bị, thực phẩm chức năng…)

1. Có thông tin về sản phẩm thử nghiệm không: Không
2. Mô tả sản phẩm nghiên cứu (không quá 1 trang): Không có đính kèm
3. Tài liệu minh chứng về sản phẩm nghiên cứu: Không có đính kèm

IV. THÔNG TIN VỀ CHIẾN LƯỢC ĐẢM BẢO ĐẠO ĐỨC NGHIÊN CỨU

1. **Lĩnh vực:** Y học lâm sàng
2. **Thiết kế nghiên cứu:** Mô tả cắt ngang, thử nghiệm lâm sàng

Mô tả biện pháp can thiệp trong nghiên cứu:

Trong thử nghiệm lâm sàng, nghiên cứu gồm 3 nhóm:

Nhóm 1: được can thiệp điều chỉnh hành vi, giáo dục bệnh nhân

Nhóm 2: điều trị laser năng lượng thấp, giáo dục bệnh nhân, điều chỉnh hành vi

Nhóm 3: điều trị bằng máng ổn định, giáo dục bệnh nhân, điều chỉnh hành vi

Mô tả biện pháp so sánh/ đối chứng

Nghiên cứu sử dụng nhóm điều trị theo phương pháp mới là laser năng lượng thấp kết hợp điều chỉnh hành vi (nhóm 2) để so sánh với nhóm điều trị theo phương pháp truyền thống bằng máng nhai kết hợp điều chỉnh hành vi (nhóm 3) và nhóm điều chỉnh hành vi đơn thuần (thay đổi thói quen, kết hợp các bài tập vận động, vật lý trị liệu chườm nóng, chườm lạnh).

3. **Đối tượng nghiên cứu:**

Mục tiêu	Đối tượng nghiên cứu	Tiêu chuẩn lựa chọn	Tiêu chuẩn loại trừ
1.Mô tả đặc điểm lâm sàng của bệnh nhân rối loạn thái dương hàm tại một số cơ sở thực hành của Viện đào tạo Răng Hàm Mặt, trường Đại học Y Hà Nội.<br><br>*2.Phân tích một số yếu tố tâm lý xã hội,*	Bệnh nhân đến khám, được chẩn đoán Rối loạn thái dương hàm (theo DC/TMD) tại ba cơ sở thực hành của Viện đào tạo Răng Hàm Mặt, Trường Đại học Y Hà Nội: Trung tâm Kỹ thuật cao RHM, Trung tâm Nha khoa 225 Trường Chinh, Khoa RHM -	Tất cả các bệnh nhân từ 16 tuổi trở lên, có tiền sử đau thoáng qua hoặc liên tục trong 3 tháng hoặc hơn, có khả năng lĩnh hội ý kiến của bác sỹ và tuân thủ quy trình khám.	Tiền sử rối loạn thần kinh nghiêm trọng, bệnh tự miễn dịch khớp và cơ, ung thư ác tính tiến triển, tiền sử bệnh tâm thần, tiền sử nghiện rượu, lạm dụng ma túy và thuốc giảm đau, bệnh nhân trải qua phẫu thuật và/ hoặc xạ trị trên vùng cổ và mặt,

hành vi của bệnh nhân rối loạn thái dương hàm.	Bệnh viện Đại học Y Hà Nội trong thời gian từ tháng 4/2023 đến tháng 10/2025.		các phương pháp điều trị khác ở vùng cổ và khớp thái dương hàm trong vòng 3 tháng, sử dụng thuốc có thể ảnh hưởng đến hệ thần kinh cơ .<br>- Bệnh nhân mang thai
3.Đánh giá kết quả của laser năng lượng thấp trong điều trị rối loạn thái dương hàm.	Bệnh nhân được chẩn đoán đau cơ thái dương và/ hoặc cơ cắn theo DC/ TMD trong nghiên cứu mô tả cắt ngang tại Trung tâm Kỹ thuật cao Răng Hàm Mặt được đưa vào nghiên cứu thử nghiệm lâm sàng.	Bệnh nhân đau cơ cấp tính (đau dưới 3 tháng)	Bệnh nhân đau TMDs mạn tính, thoái hoá khớp, sưng đau trong miệng cản trở việc đeo máng nhai.

4. **Tuổi giới hạn**

Mục tiêu	Đối tượng nghiên cứu	Tuổi thấp nhất	Tuổi cao nhất
1.Mô tả đặc điểm lâm sàng của bệnh nhân rối loạn thái dương hàm tại một số cơ sở thực hành của Viện đào tạo Răng Hàm Mặt, trường Đại học Y Hà Nội.	Bệnh nhân đến khám, được chẩn đoán Rối loạn thái dương hàm	16	Không giới hạn

2.Phân tích một số yếu tố tâm lý xã hội, hành vi của bệnh nhân rối loạn thái dương hàm.			
3.Đánh giá kết quả của laser năng lượng thấp trong điều trị rối loạn thái dương hàm.	Bệnh nhân được chẩn đoán đau cơ thái dương và/ hoặc cơ cắn cấp tính	16	Không giới hạn

5. Có tuyển đối tượng nghiên cứu là phụ nữ hay không?
Có
Nếu CÓ, ước lượng tỷ lệ phụ nữ tham gia nghiên cứu: 60%
Nếu KHÔNG, nêu rõ lý do:

6. Có tuyển đối tượng nghiên cứu là trẻ em hay không?
Không
Nếu CÓ, ước lượng tỷ lệ trẻ em tham gia nghiên cứu:
Nếu KHÔNG, nêu rõ lý do: tỷ lệ trẻ em rối loạn thái dương hàm được báo cáo không nhiều.

7. Cỡ mẫu nghiên cứu:

**Mục tiêu**	**Đối tượng nghiên cứu**	**Cỡ mẫu dự kiến**
1.Mô tả đặc điểm lâm sàng của bệnh nhân rối loạn thái dương hàm tại một số cơ sở thực hành của Viện đào tạo Răng Hàm Mặt, trường Đại học Y Hà Nội.<br><br>*2.Phân tích một số yếu tố tâm lý xã hội, hành vi của bệnh nhân rối loạn thái dương hàm.*	Bệnh nhân đến khám, được chẩn đoán Rối loạn thái dương hàm	120
3.Đánh giá kết quả của laser năng lượng thấp trong điều trị rối loạn thái dương hàm.	Bệnh nhân được chẩn đoán đau cơ thái dương và/ hoặc cơ cắn cấp hoặc bán cấp	66

8. Nguồn dữ liệu thu thập:

Mục tiêu	Nguồn thu thập/ Cách thức thu thập	Số lượng/ Khối lượng
1.Mô tả đặc điểm lâm sàng của bệnh nhân rối loạn thái dương hàm tại một số cơ sở thực hành của Viện đào tạo Răng Hàm Mặt, trường Đại học Y Hà Nội.<br><br>*2.Phân tích một số yếu tố tâm lý xã hội, hành vi của bệnh nhân rối loạn thái dương hàm.*	Thu thập thông tin bằng cách sử dụng bộ câu hỏi phỏng vấn bệnh nhân, bác sỹ thăm khám lâm sàng ghi nhận các triệu chứng lâm sàng vào bệnh án nghiên cứu	120
3.Đánh giá kết quả của laser năng lượng thấp trong điều trị rối loạn thái dương hàm.	Thu thập thông tin bằng cách sử dụng bộ câu hỏi phỏng vấn bệnh nhân, bác sỹ thăm khám lâm sàng ghi nhận các triệu chứng lâm sàng vào bệnh án nghiên cứu	66

9. Quy trình tuyển chọn và duy trì đối tượng tham gia nghiên cứu: Có đính kèm

10. Tình trạng tuyển chọn người tham gia nghiên cứu: Chưa bắt đầu

11. Dự kiến thời gian tuyển chọn người đầu tiên: 1/04/2023

12. Khung thời gian tuyển chọn người tham gia nghiên cứu: từ tháng 4 năm 2023 đến tháng 10 năm 2025

13. Những hoạt động nghiên cứu mà người tham gia phải thực hiện nếu đồng ý tham gia vào nghiên cứu là gì?

Người tham gia nghiên cứu cần một ít thời gian để hỏi và hiểu rõ về nghiên cứu, nghe và trả lời những câu hỏi liên quan đến bệnh lý của mình. Người tham gia sẽ được bác sỹ thăm khám để ghi nhận những triệu chứng lâm sàng, chụp phim MRI hoặc CT Cone Beam tuỳ theo chỉ định và giai đoạn bệnh của bệnh lý.

Trong thử nghiệm lâm sàng, người tham gia cần phối hợp với bác sỹ tuân thủ chặt chẽ quy trình điều trị, các bài tập vận động, thay đổi thói quen có hại cho khớp thái dương hàm, đồng thời tái khám chính xác theo đúng lịch hẹn của bác sỹ.

14. Những nguy cơ người tham gia nghiên cứu có thể gặp phải là gì?

Bệnh nhân có thể gặp một chút khó chịu trong quá trình điều trị. Nhưng việc này là bình thường và sẽ hết nhanh sau đó.

Nếu không tham gia nghiên cứu, bệnh nhân và gia đình sẽ tự chi trả khoản chi phí này.

15. Những biện pháp hạn chế/ giảm thiểu rủi ro và tăng cường lợi ích cho đối tượng khi tham gia nghiên cứu là gì?

Bệnh nhân sẽ được hướng dẫn, theo dõi kỹ lưỡng trước, trong và sau nghiên cứu. Khi có bất kỳ vấn đề nào phát sinh, bệnh nhân sẽ được hướng dẫn liên lạc ngay với bác sỹ điều trị để kịp thời giải quyết và khắc phục.

16. Mô tả tiến trình chấp thuận tham gia nghiên cứu và duy trì đối tượng tham gia nghiên cứu:

Bệnh nhân đến khám được chẩn đoán Rối loạn thái dương hàm tại ba cơ sở thực hành của Viện đào tạo Răng Hàm Mặt, Trường Đại học Y Hà Nội trong thời gian nghiên cứu sẽ được mời tham gia vào nghiên cứu. Quyết định tham gia là hoàn toàn tự nguyện, không ai có thể ép buộc hoặc dụ dỗ. Bệnh nhân có thể từ chối, thay đổi quyết định hay rút khỏi nghiên cứu mà không lo ngại ảnh hưởng đến quy trình thăm khám và chữa bệnh thường quy tại bệnh viện.

Sau khi được cung cấp thông tin về nghiên cứu, những lợi ích và bất lợi dưới đây, bệnh nhân có thể hỏi thành viên trong nhóm nghiên cứu bất kỳ câu hỏi nào nếu còn thắc mắc. Nếu đồng ý tham gia nghiên cứu, bệnh nhân ký tên vào bản đồng thuận.

Sau khi được phỏng vấn bằng bộ câu hỏi, thăm khám lâm sàng, một phần bệnh nhân sẽ tham gia tiếp vào thử nghiệm lâm sàng. Các bệnh nhân này sẽ được hướng dẫn cách ghi báo cáo vào nhật ký theo dõi hàng ngày, quay video và gửi cho bác sỹ (các bài tập vận động, vật lý trị liệu, thay đổi thói quen miệng có hại). Bệnh nhân sẽ được hẹn tái khám sau 2 tuần, 4 tuần, 12 tuần, 24 tuần, 48 tuần để kiểm tra mức độ cải thiện triệu chứng lâm sàng và tái phát. Trước mỗi lần hẹn, nhân viên y tế sẽ gọi điện nhắc trước 1 ngày để bệnh nhân sắp xếp, tránh sai hoặc bỏ lỡ lịch khám lại.

Những thông tin về sức khoẻ của bệnh nhân sẽ được giữ bảo mật.

17. Biện pháp để đảm bảo an toàn dữ liệu nghiên cứu là gì?

Hồ sơ bệnh án của bệnh nhân sẽ được dán mã số nghiên cứu và được kiểm tra thường xuyên bởi nhóm nghiên cứu. Tên của bệnh nhân sẽ không được sử dụng trong các báo cáo cũng như các công bố khoa học liên quan. Các thông tin khoa học khác như phim Xquang, bệnh án nghiên cứu, phiếu trả lời câu hỏi sẽ được lưu trữ an toàn và chỉ được sử dụng khi được phép của Hội đồng y đức và người tham gia nghiên cứu.

18. Bản cung cấp thông tin cho đối tượng tham gia nghiên cứu? Có đính kèm
