# Supplementary material for: Photobiomodulation versus stabilization splint, both combined with self-management, for acute temporomandibular myalgia: 48-week outcomes of a partially randomized controlled study
Source: PLoS One. 2026 Oct 27;21(10):e0360472. doi: 10.1371/journal.pone.0360472 (PMC13712594; doi:10.1371/journal.pone.0360472)
Supplement: Supplementary file 2 [file pone.0360472.s004.pdf]

HANOI MEDICAL UNIVERSITY
INSTITUTIONAL ETHICAL REVIEW BOARD

RESEARCH PROTOCOL

CLINICAL, PSYCHOSOCIAL CHARACTERISTICS AND ORAL BEHAVIORS AND EFFECTIVENESS OF LOW – LEVEL LASER THERAPY IN TEMPOROMANDIBULAR DISORDER PATIENTS

Principal Investigator: Nguyen Ngoc Hoa

Affiliation: Hanoi Medical University

Protocol Version: 1.0

Protocol Date: 12 January 2023

Study Period: 2023–2025

PRINCIPAL INVESTIGATOR
(Signature and full name)

Nguyen Ngoc Hoa

Hanoi, 2023

I. GENERAL PROJECT INFORMATION

1. **Submission Status:** New submission
2. **Review Type:** Initial review
3. **Project Title:** Clinical, psychosocial characteristics and oral behaviors and effectiveness of low-level laser therapy in temporomandibular disorder patients
4. **Project Code:** NCS
5. **Dossier ID:** 858
6. **Principal Investigator (PI) Information:**
    - Full Name: Nguyen Ngoc Hoa
    - Gender: Female
    - Date of Birth:
    - Position: Lecturer
    - Affiliation: Hanoi Medical University
    - Academic Rank: None
    - Academic Degree: Medical Resident
    - Phone:
    - Email: nguyenngochoa@hmu.edu.vn
7. **Host Institution:** Hanoi Medical University
    - 7.1 Address: No. 1 Ton That Tung, Dong Da, Hanoi
    - 7.2 Representative: Nguyen Huu Tu
    - 7.3 Position: Rector
    - 7.4 Phone:
    - 7.5 Email: nguyenhuutu@hmu.edu.vn
8. **Funding Body / Sponsor:** None
9. **Total Budget:** 135,700,000 VND
10. **Start Date:** April 2023 | **End Date:** October 2025
11. **Project Classification:** Doctoral (PhD) Research Project
12. **Project Secretary:**
    - Account Name: nguyenngochoa@hmu.edu.vn
    - Full Name: Nguyen Ngoc Hoa
    - Academic Rank: None
    - Academic Degree: Medical Resident
    - Affiliation: Hanoi Medical University
    - Email: nguyenngochoa@hmu.edu.vn
    - Phone:
13. **Ethics Committee Review History:**

**Review Round**	**Outcome**	**Approval No.**	**Date of Approval**	**Dossier Version**
1	Approved	858	March 14, 2026	1

14. **Application Form:** Attached

II. ETHICAL APPROVAL CERTIFICATE INFORMATION

In Vietnamese:

- **Project Title:** Clinical, psychosocial characteristics and oral behaviors and effectiveness of low-level laser therapy in temporomandibular disorder patients
- **Principal Investigator (PI):** Nguyen Ngoc Hoa
- **Sponsor / Funding Body:** None
- **Host Institution:** Hanoi Medical University
- **Study Sites:** Clinical practice facilities of the School of Dentistry - Hanoi Medical University, including: the High-Tech Center for Odonto-Stomatology, the 225 Truong Chinh Dental Center, and the Department of Odonto-Stomatology at Hanoi Medical University Hospital.
- **Start Date:** April 1, 2023 | **End Date:** October 31, 2025
- **List of documents reviewed and approved by the Ethics Committee:**

No.	Document Title	Version No.	Date
1	Detailed Research Protocol	01	20/01/2023
2	Case Report Form (CRF)	01	20/01/2023
3	Informed Consent Form (ICF) for Cross-Sectional Study	01	20/01/2023
4	Informed Consent Form (ICF) for Clinical Trial	01	20/01/2023

RESEARCH PROTOCOL

Project Title: Clinical, psychosocial characteristics and oral behaviors and effectiveness of low-level laser therapy in temporomandibular disorder patients

Introduction and Rationale: Temporomandibular Disorders (TMDs) are recognized as a cluster of musculoskeletal conditions involving the masticatory muscles, the temporomandibular joint (TMJ), and associated structures. They represent the primary cause of non-odontogenic pain in the orofacial region.

Symptoms of TMDs severely impair patient function and activities of daily living, leading to a diminished quality of life if left untreated or inappropriately managed. Epidemiological studies indicate that approximately 10% to 15% of the general population are affected by TMDs, with around 5% of surveyed individuals requiring treatment. In Vietnam, the prevalence of symptomatic TMD patients varies widely, ranging from 20.6% to 60.5% across different studies. These findings demonstrate that while prevalence and symptomatology may vary across populations, the demand for TMD treatment remains substantial worldwide, and particularly in Vietnam.

Given the multifactorial etiology of TMDs, therapeutic approaches are broad and diverse. Numerous recent studies recommend conservative, non-invasive, and reversible interventions. These include pharmacological therapies as well as non-pharmacological modalities such as occlusal splints, dry needling, physical therapy and rehabilitation, ultrasound therapy, transcutaneous electrical nerve stimulation (TENS), relaxation techniques, acupuncture, stretching exercises, massage therapy, low-level laser therapy (LLLT), and psychological interventions. LLLT has been demonstrated to be safe, virtually free of adverse effects, cost-effective, requiring a limited number of sessions, and straightforward to administer.

In Vietnam, however, there is currently a lack of published research evaluating the therapeutic efficacy of LLLT, and its clinical application remains restricted to a limited number of major medical centers. The widespread adoption of this modality could offer substantial therapeutic benefits to patients by alleviating pain, improving maximum mouth opening, and enhancing mandibular mobility.

In the present study, we will investigate the clinical, psychosocial, and behavioral characteristics of 120 patients with TMDs presenting at three clinical practice facilities of the School of Odonto-Stomatology, Hanoi Medical University. Furthermore, 66 patients at the High-Tech Odonto-Stomatology Center, Hanoi Medical University, will be recruited to participate in a clinical trial designed to evaluate the therapeutic efficacy of low-level laser therapy in the management of temporomandibular disorders.

Research Objectives

1. To describe the clinical characteristics of patients with temporomandibular disorders (TMDs) at selected clinical practice facilities of the School of Odonto-Stomatology, Hanoi Medical University.
2. To analyze specific psychosocial and behavioral factors among patients with temporomandibular disorders.
3. To evaluate the efficacy of low-level laser therapy (LLLT) in the treatment of temporomandibular disorders.

Study Population

Our study consists of two phases: a descriptive cross-sectional study and a controlled clinical trial.

<u>In the Descriptive Cross-Sectional Study:</u>

Inclusion Criteria:

- Patients presenting for examination and diagnosed with temporomandibular disorders at three clinical practice facilities of the School of Odonto-Stomatology, Hanoi Medical University (the High-Tech Odonto-Stomatology Center, the 225 Truong Chinh Dental Center, and the Department of Odonto-Stomatology at Hanoi Medical University Hospital) between April 2023 and October 2025.
- Patients aged 16 years or older.

- Patients who are fully capable of understanding the physician's instructions and complying with the clinical examination protocol.

Exclusion Criteria:

- Patients with a history of severe neurological disorders, musculoskeletal autoimmune diseases, or progressive malignancies.
- Patients with a history of psychiatric disorders, alcoholism, substance abuse, or analgesic abuse.
- Patients who have undergone surgery and/or radiation therapy in the face and neck region, or received other therapeutic modalities involving the neck region and the temporomandibular joint within the past 3 months.
- Patients currently using medications that could affect the neuromuscular system.
- Pregnant patients.

<u>In the Controlled Clinical Trial:</u>

Patients will be recruited in parallel from the descriptive cross-sectional study at the High-Tech Odonto-Stomatology Center, provided they give informed consent to participate in the study.

- **Inclusion Criteria:** Patients diagnosed with acute pain of the temporalis and/or masseter muscles (pain duration < 3 months) and a painless maximum mouth opening (MMO) of less than 40 mm.
- **Exclusion Criteria:** Patients with chronic pain (pain duration of 3 months or longer), temporomandibular joint osteoarthritis, or intraoral pain and swelling that interferes with the use of an occlusal splint.

Study Period and Setting:

From April 2023 to October 2025 at the High-Tech Odonto-Stomatology Center, Hanoi Medical University.

Study Methodology (Sample Sizes):

- **Descriptive cross-sectional study:** Sample size of 120 patients.
- **Controlled clinical trial:** Sample size of 66 patients.

Study Procedures:
For the Descriptive Cross-Sectional Study:

- **Interview and Questionnaire Administration:** Upon presenting for clinical examination regarding temporomandibular disorders, patients will be interviewed to collect demographic and administrative data (full name, age, sex, address, telephone number, and chief complaint). Patients will then receive data collection instruments with standardized guidance from the clinician. These instruments comprise: a symptom questionnaire, a Visual Analog Scale (VAS) for pain assessment, a chronic pain grading scale, a mandibular

function limitation questionnaire, depression and anxiety screening scales, an oral parafunctional habits questionnaire, and a health-related quality of life (QoL) questionnaire. Patients will complete these questionnaires during their initial clinical visit.
- **Clinical Examination:** The clinician will perform a standardized clinical examination of the temporomandibular joint and its associated structures following established protocols. Full patient cooperation during the examination is required to ensure optimal diagnostic accuracy.
- **Diagnostic Imaging:** Magnetic Resonance Imaging (MRI) or Cone Beam Computed Tomography (CBCT) will be indicated based on the patient's specific clinical presentation to establish a definitive diagnosis.
- **Quality Control and Supervision:** The entire process of interviewing, initial clinical examination, and follow-up visits will be conducted by the principal investigator under the direct supervision of the research advisor.

For the Controlled Clinical Trial

Patients meeting the inclusion criteria will receive treatment counseling. Among those who decline the active interventions due to financial constraints, we will seek to recruit them into the control group (Group 1). Group 1 will comprise 22 patients who will undergo behavioral modification; these individuals will be enrolled consecutively based on their presentation order until the target sample size is reached. The 44 patients who consent to active treatment will be randomized into the remaining two groups. Randomization will be performed using Microsoft Excel to generate a random number sequence. Allocation details will then be placed in sequentially numbered, sealed, opaque envelopes across the three study sites (this step will be executed by an independent individual not directly involved in the core research team).

Once a patient meets all eligibility criteria (inclusion and exclusion) and provides written informed consent, a clinician from the research team will open the envelope corresponding to the predefined sequential number and allocate the participant according to the following protocol: even numbers will be assigned to Group 2, and odd numbers to Group 3.

- **Group 2:** Low-level laser therapy (LLLT), patient education, and behavioral modification.
- **Group 3:** Stabilization splint therapy, patient education, and behavioral modification.

Patients will be instructed to strictly adhere to home-based physical exercises and self-therapy regimens, alongside behavioral modifications aimed at altering detrimental habits. These interventions will focus on head, neck, and shoulder posture, as well as sleep and dietary adjustments.

Our research hypothesis is: *"In patients with temporomandibular disorders, two multimodal treatment approaches—low-level laser therapy combined with behavioral modification, and occlusal splint therapy combined with behavioral modification—will both yield longitudinal improvements in clinical outcomes, functional status, and patient-reported perceptions. The study primarily focuses on comparing the efficacy between these two randomized treatment arms. Additionally, the outcomes of these two multimodal strategy groups will be compared with those of the behavioral modification-only control group."*

Following treatment, patients will be scheduled for follow-up visits at 2, 4, 12, 24, and 48 weeks to undergo:

- **Interviews and Questionnaires:** Visual Analog Scale (VAS) for pain assessment, the 8-item Jaw Functional Limitation Scale (JFLS-8), and post-treatment global improvement ratings.
- **Clinical Re-examination:** Muscle tenderness scores, painless maximum mouth opening (MMO), joint sounds (clicks/crepitus), and lateral as well as protrusive mandibular excursions.

DATA MANAGEMENT AND ANALYSIS

Outcomes will be assessed between the randomized groups (LLLT+SM vs. OS+SM) under an intention-to-treat principle. Repeated measures at baseline (T0) and weeks 2, 4, 12, 24, and 48 will be analyzed using linear mixed-effects models. A p-value < 0.05 will be considered statistically significant.

Expected Research Outcomes

1. To describe the clinical characteristics of patients with temporomandibular disorders (TMDs) at selected clinical practice facilities of the School of Odonto-Stomatology, Hanoi Medical University.
2. To analyze specific psychosocial and behavioral factors among patients with temporomandibular disorders.
3. To evaluate the efficacy of low-level laser therapy (LLLT) in the treatment of temporomandibular disorders.

III. INVESTIGATIONAL PRODUCT INFORMATION

(Medicines, medical devices, dietary supplements...)

1. **Is there information available on the investigational product?** No
2. **Investigational product description (maximum 1 page):** Not attached
3. **Supporting documentation for the investigational product:** Not attached

IV. ETHICAL ASSURANCE STRATEGY IN RESEARCH

1. **Research Field:** Clinical medicine
2. **Study Design:** Cross-sectional descriptive study, clinical trial

Description of Research Interventions:

In the clinical trial, the study population is divided into 3 groups:

- **Group 1:** Interventions consist of behavioral modification and patient education.
- **Group 2:** Low-level laser therapy (LLLT), patient education, and behavioral modification.

- **Group 3:** Stabilization splint therapy, patient education, and behavioral modification.

Description of Comparators / Controls:

The study utilizes the novel treatment strategy—consisting of low-level laser therapy combined with behavioral modification (Group 2)—to compare against the traditional treatment strategy using an occlusal splint combined with behavioral modification (Group 3), as well as a behavioral modification-only control group (which involves habit modification, physical exercises, and physical therapy utilizing hot/cold compresses).

3. Study Population

Objective	Study Population	Inclusion Criteria	Exclusion Criteria
**1. To describe the clinical characteristics** of patients with temporomandibular disorders (TMDs) at selected clinical practice facilities of the School of Odonto-Stomatology, Hanoi Medical University.<br><br>**2. To analyze specific psychosocial and behavioral factors** among patients with temporomandibula-r disorders.	Patients presenting for clinical examination and diagnosed with temporomandibular disorders (according to the **DC/TMD** criteria) at three clinical practice facilities of the School of Odonto-Stomatology, Hanoi Medical University (the High-Tech Odonto-Stomatology Center, the 225 Truong Chinh Dental Center, and the Department of Odonto-Stomatology at Hanoi Medical University Hospital) between April 2023 and October 2025.	* All patients aged 16 years or older.<br><br>* History of transient or continuous pain within the past 3 months or longer.<br><br>* Capable of understanding the physician's instructions and complying with the clinical examination protocol.	* History of severe neurological disorders, musculoskeletal autoimmune diseases, or progressive malignancies.<br><br>* History of psychiatric disorders, alcoholism, substance abuse, or analgesic abuse.<br><br>* Patients who have undergone surgery and/or radiation therapy in the face and neck region, or received other therapeutic modalities involving the neck region and the temporomandibular joint within the past 3 months.

Objective	Study Population	Inclusion Criteria	Exclusion Criteria
			* Current use of medications that could affect the neuromuscular system.<br><br>* Pregnant patients.
**3. To evaluate the efficacy** of low-level laser therapy (LLLT) in the treatment of temporomandibular disorders.	Patients diagnosed with pain of the temporalis and/or masseter muscles according to the **DC/TMD** criteria within the descriptive cross-sectional study at the Center [High-Tech Odonto-Stomatology Center].	Patients with acute muscle pain (pain duration < 3 months).	* Patients with chronic TMD pain (pain duration $\ge$ 3 months).<br><br>* Temporomandibular joint osteoarthritis.<br><br>* Intraoral pain and swelling that interferes with the use of an occlusal splint.

4. Age Limits

Objective	Study Population	Minimum Age	Maximum Age
**1. To describe the clinical characteristics** of patients with temporomandibular disorders (TMDs) at selected clinical practice facilities of the School of Odonto-Stomatology, Hanoi Medical University.	Patients presenting for clinical examination and diagnosed with temporomandibular disorders.	16	No upper limit

Objective	Study Population	Minimum Age	Maximum Age
**2. To analyze specific psychosocial and behavioral factors** among patients with temporomandibular disorders.			
**3. To evaluate the efficacy** of low-level laser therapy (LLLT) in the treatment of temporomandibular disorders.	Patients diagnosed with acute pain of the temporalis and/or masseter muscles.	16	No upper limit

5. Does the study recruit female participants?

- Yes
- If YES, estimated proportion of female participants: 60%
- If NO, state the reason:

6. Does the study recruit children/pediatric participants?

- No
- If YES, estimated proportion of children/pediatric participants:
- If NO, state the reason: The reported prevalence of pediatric patients with temporomandibular disorders is low.

7. Sample size

Objective	Study Population	Target Sample Size
**1.** To describe the clinical characteristics of patients with temporomandibular disorders (TMDs) at selected clinical practice facilities of the School of Odonto-Stomatology, Hanoi Medical University.<br><br>**2.** To analyze specific psychosocial and behavioral factors among patients with temporomandibular disorders.	Patients presenting for clinical examination and diagnosed with temporomandibular disorders.	120

Objective	Study Population	Target Sample Size
**3.** To evaluate the efficacy of low-level laser therapy (LLLT) in the treatment of temporomandibular disorders.	Patients diagnosed with acute or subacute pain of the temporalis and/or masseter muscles.	66

8. Data Collection Sources

Objective	Data Source / Collection Method	Quantity / Volume
**1.** To describe the clinical characteristics of patients with temporomandibular disorders (TMDs) at selected clinical practice facilities of the School of Odonto-Stomatology, Hanoi Medical University.<br><br>**2.** To analyze specific psychosocial and behavioral factors among patients with temporomandibular disorders.	Data will be collected using patient interview questionnaires. Clinicians will perform clinical examinations and record clinical symptoms into the Case Report Form (CRF).	120
**3.** To evaluate the efficacy of low-level laser therapy (LLLT) in the treatment of temporomandibular disorders.	Data will be collected using patient interview questionnaires. Clinicians will perform clinical examinations and record clinical symptoms... *(văn bản gốc bị cắt ngang ở dòng này)*	66

9. **Participant recruitment and retention procedures:** Attached.
10. **Participant recruitment status:** Not yet started.
11. **Expected date of first participant recruitment:** April 1, 2023.
12. **Participant recruitment timeframe:** From April 2023 to October 2025.
13. **Research activities required of participants:**

Participants will be allotted time to inquire and gain a clear understanding of the study, as well as to pose questions regarding their condition.

Participants will undergo clinical examinations to record clinical symptoms and may require Magnetic Resonance Imaging (MRI) or Cone Beam Computed Tomography (CBCT), contingent upon the specific clinical indication and stage of the disease.

In the clinical trial, participants must cooperate with the physician, adhere strictly to treatment protocols, physical exercises, and modifications of habits detrimental to the temporomandibular joint, as well as comply with scheduled follow-up appointments.

14. Potential risks to participants

Patients may experience minor discomfort during the treatment process; however, this is considered normal and transient.

If they choose not to participate in the study, patients and their families will be responsible for bearing these costs themselves.

15. Risk mitigation and benefit enhancement measures

Patients will be guided and monitored closely before, during, and after the study.Should any issues arise, patients will be instructed to contact the attending physician immediately for timely resolution and management.

16. Description of Informed Consent and Participant Retention Process (Continued):

- Patients presenting for examination and diagnosed with temporomandibular disorders at the three clinical practice facilities of the School of Odonto-Stomatology, Hanoi Medical University during the study period will be invited to participate in the study. Participation is entirely voluntary; no form of coercion or inducement is permitted. Patients retain the right to refuse, alter their decision, or withdraw from the study at any time without any concern regarding the impact on their standard care and routine clinical services at the hospital.
- After being provided with information regarding the study, including the benefits and disadvantages outlined below, patients may ask the research team members any remaining questions they may have. If they agree to participate in the study, patients will sign the Informed Consent Form (ICF).
- Following the questionnaire-based interview and clinical examination, a subset of patients will participate in the clinical trial. These patients will be instructed on how to record data in a daily monitoring log, film videos, and submit them to the physician (covering physical exercises, physical therapy, and oral parafunctional habit modifications). Patients will be scheduled for follow-up visits at 2, 4, 12, 24, and 48 weeks to evaluate the degree of clinical symptom improvement and recurrence. Prior to each scheduled appointment, healthcare staff will call to remind the patient 1 day in advance, allowing them to arrange their schedule and avoid missing or skipping the follow-up visit.
- The patient's health information will be kept strictly confidential.

17. Measures to Ensure Research Data Security

- The patients' medical records will be coded with unique research identification numbers and regularly reviewed by the research team. Patients' names will not be used in any reports

or related scientific publications. Other scientific data—such as X-rays, research case records, and completed questionnaires—will be stored securely and utilized only upon authorization by the Ethics Committee and the research participants.

18. Participant Information Sheet? Attached.
