# Supplementary material for: Photobiomodulation versus stabilization splint, both combined with self-management, for acute temporomandibular myalgia: 48-week outcomes of a partially randomized controlled study
Source: PLoS One. 2026 Oct 27;21(10):e0360472. doi: 10.1371/journal.pone.0360472 (PMC13712594; doi:10.1371/journal.pone.0360472)
Supplement: Supplementary file 3 [file pone.0360472.s001.docx]

**Table S1. Primary outcomes over time by treatment group (Median (IQR)).**

| **Outcome** | **Time** | | **SM Group** | **PBM+SM Group** | **OS+SM Group** |
| --- | --- | --- | --- | --- | --- |
| **VAS (0–10)** | | | | | |
|  |  | T0 | 5.42 (4.95–5.84); n=22 | 5.50 (5.08–6.09); n=22 | 6.37 (5.56–6.60); n=22 |
|  | **Early response** | T2 | 4.56 (4.10–5.15); n=22 | 3.33 (2.55–4.42); n=22 | 3.83 (3.39–4.26); n=22 |
|  |  | T4 | 4.29 (3.77–4.68); n=22 | 0.89 (0.42–1.43); n=22 | 1.41 (1.15–2.23); n=22 |
|  | **Mid-term** | T12 | 3.92 (3.60–4.66); n=22 | 0.12 (0.00–0.60); n=22 | 0.41 (0.01–0.56); n=22 |
|  |  | T24 | 2.68 (1.67–3.42); n=20 | 0.00 (0.00–0.35); n=20 | 0.00 (0.00–0.33); n=22 |
|  | **Long-term** | T48 | 0.96 (0.65–1.88); n=19 | 0.00 (0.00–0.13); n=19 | 0.00 (0.00–0.00); n=21 |
| **Maximum painless mouth opening (MPMO, mm)** | | | | | |
|  |  | T0 | 30.00 (30.00–35.00); n=22 | 30.00 (25.00–35.00); n=22 | 30.00 (25.00–35.00); n=22 |
|  | **Early response** | T2 | 32.00 (30.25–36.00); n=22 | 32.00 (29.00–37.75); n=22 | 33.50 (30.50–38.00); n=22 |
|  |  | T4 | 34.00 (30.00–36.75); n=22 | 35.50 (32.25–41.75); n=22 | 36.50 (35.00–40.00); n=22 |
|  | **Mid-term** | T12 | 34.50 (32.00–37.00); n=22 | 40.50 (37.50–42.75); n=22 | 40.00 (38.00–42.75); n=22 |
|  |  | T24 | 38.00 (36.00–42.25); n=20 | 45.00 (41.75–45.00); n=20 | 45.00 (41.00–47.00); n=22 |
|  | **Long-term** | T48 | 41.00 (38.50–43.50); n=19 | 47.00 (45.00–49.00); n=19 | 48.00 (46.00–50.00); n=21 |
| **Number of masticatory muscle palpation-tender points** | | | | | |
|  |  | T0 | 3.00 (2.00–4.00); n=22 | 3.00 (2.00–4.00); n=22 | 3.00 (2.00–5.50); n=22 |
|  | **Early response** | T2 | 3.00 (2.00–3.75); n=22 | 2.00 (2.00–3.00); n=22 | 2.00 (2.00–3.75); n=22 |
|  |  | T4 | 2.00 (2.00–3.00); n=22 | 1.00 (1.00–2.00); n=22 | 1.00 (1.00–2.75); n=22 |
|  | **Mid-term** | T12 | 2.00 (2.00–3.00); n=22 | 0.00 (0.00–1.00); n=22 | 1.00 (0.00–1.00); n=22 |
|  |  | T24 | 1.00 (0.00–2.00); n=20 | 0.00 (0.00–0.00); n=20 | 0.00 (0.00–0.00); n=22 |
|  | **Long-term** | T48 | 0.00 (0.00–1.00); n=19 | 0.00 (0.00–0.00); n=19 | 0.00 (0.00–0.00); n=21 |

### *Values are median (IQR); n is the number of participants with available data at each time point. T0 baseline, T2, T4, T12, T24, and T48 follow-up (weeks). MPMO: maximum painless mouth opening; VAS: Visual Analogue Scale.*

**Table S2. Primary outcomes over time (Mean (SD))**

| **Outcome** | **Time** | **SM Group** | **PBM+SM Group** | **OS+SM Group** |
| --- | --- | --- | --- | --- |
| **VAS (0–10)** | | | | |
|  | **T0** | 5.47 (0.90); n=22 | 5.73 (1.32); n=22 | 6.12 (0.87); n=22 |
|  | **T2** | 4.62 (0.69); n=22 | 3.40 (1.24); n=22 | 3.77 (0.94); n=22 |
|  | **T4** | 4.42 (0.89); n=22 | 1.19 (1.07); n=22 | 1.66 (1.19); n=22 |
|  | **T12** | 4.16 (0.85); n=22 | 0.50 (0.82); n=22 | 0.44 (0.52); n=22 |
|  | **T24** | 2.69 (1.24); n=20 | 0.21 (0.44); n=20 | 0.42 (1.32); n=22 |
|  | **T48** | 1.43 (1.23); n=19 | 0.09 (0.18); n=19 | 0.28 (1.04); n=21 |
| **Maximum mouth painless opening (MMO, mm)** | | | | |
|  | **T0** | 31.36 (6.02); n=22 | 29.95 (7.05); n=22 | 30.32 (7.72); n=22 |
|  | **T2** | 33.00 (5.42); n=22 | 33.00 (5.48); n=22 | 34.09 (6.41); n=22 |
|  | **T4** | 33.73 (5.45); n=22 | 36.45 (4.71); n=22 | 36.68 (6.32); n=22 |
|  | **T12** | 34.77 (5.29); n=22 | 40.18 (3.84); n=22 | 40.50 (5.51); n=22 |
|  | **T24** | 38.30 (4.81); n=20 | 43.30 (3.29); n=20 | 43.82 (4.69); n=22 |
|  | **T48** | 40.63 (4.31); n=19 | 46.16 (3.24); n=19 | 47.33 (4.13); n=21 |
| **Number of masticatory muscle palpation-tender points** | | | | |
|  | **T0** | 3.23 (1.45); n=22 | 3.41 (1.59); n=22 | 4.00 (2.43); n=22 |
|  | **T2** | 3.00 (1.31); n=22 | 2.59 (1.33); n=22 | 2.82 (1.68); n=22 |
|  | **T4** | 2.68 (1.43); n=22 | 1.32 (0.84); n=22 | 1.86 (1.52); n=22 |
|  | **T12** | 2.36 (1.40); n=22 | 0.41 (0.67); n=22 | 0.91 (1.51); n=22 |
|  | **T24** | 1.25 (1.48); n=20 | 0.15 (0.37); n=20 | 0.36 (1.50); n=22 |
|  | **T48** | 0.84 (1.42); n=19 | 0.00 (0.00); n=19 | 0.33 (1.53); n=21 |

*Values are mean (SD). n denotes participants with available data at each time point. T0 baseline; T2–T48 follow-up (weeks). SM was a non-randomized convenience comparator. MMO, maximum mouth painless opening; VAS, visual analogue scale.*

**Table S3. Model-based between-group contrasts over time (T2–T48)**

| **Outcome** | **Time** | **Comparison** | **Effect type** | **Estimate** | **95% CI** | **p-value** | **p (Holm)** | **p (FDR)** |
| --- | --- | --- | --- | --- | --- | --- | --- | --- |
| **VAS** | **T2** | **PBM vs OS** | MD | -0.37 | -0.94 to 0.20 | 0.203 | 0.811 | 0.253 |
|  |  | **PBM vs SM*** | MD | -1.22 | -1.79 to -0.65 | <0.001 | <0.001 | <0.001 |
|  |  | **OS vs SM*** | MD | -0.85 | -1.42 to -0.28 | 0.004 | 0.022 | 0.005 |
|  | **T4** | **PBM vs OS** | MD | -0.47 | -1.04 to 0.10 | 0.108 | 0.540 | 0.147 |
|  |  | **PBM vs SM*** | MD | -3.23 | -3.80 to -2.66 | <0.001 | <0.001 | <0.001 |
|  |  | **OS vs SM*** | MD | -2.76 | -3.34 to -2.19 | <0.001 | <0.001 | <0.001 |
|  | **T12** | **PBM vs OS** | MD | 0.06 | -0.51 to 0.63 | 0.836 | 0.873 | 0.836 |
|  |  | **PBM vs SM*** | MD | -3.66 | -4.23 to -3.09 | <0.001 | <0.001 | <0.001 |
|  |  | **OS vs SM*** | MD | -3.72 | -4.29 to -3.15 | <0.001 | <0.001 | <0.001 |
|  | **T24** | **PBM vs OS** | MD | -0.31 | -0.89 to 0.27 | 0.291 | 0.873 | 0.323 |
|  |  | **PBM vs SM*** | MD | -2.50 | -3.08 to -1.91 | <0.001 | <0.001 | <0.001 |
|  |  | **OS vs SM*** | MD | -2.18 | -2.76 to -1.60 | <0.001 | <0.001 | <0.001 |
|  | **T48** | **PBM vs OS** | MD | -0.31 | -0.90 to 0.28 | 0.301 | 0.873 | 0.323 |
|  |  | **PBM vs SM*** | MD | -1.31 | -1.91 to -0.71 | <0.001 | <0.001 | <0.001 |
|  |  | **OS vs SM*** | MD | -1.00 | -1.59 to -0.41 | <0.001 | 0.006 | 0.001 |
| **MPMO** | **T2** | **PBM vs OS** | MD | -1.09 | -4.20 to 2.02 | 0.492 | 1.000 | 0.671 |
|  |  | **PBM vs SM*** | MD | -0.00 | -3.11 to 3.11 | 1.000 | 1.000 | 1.000 |
|  |  | **OS vs SM*** | MD | 1.09 | -2.02 to 4.20 | 0.492 | 1.000 | 0.671 |
|  | **T4** | **PBM vs OS** | MD | -0.23 | -3.34 to 2.89 | 0.886 | 1.000 | 0.950 |
|  |  | **PBM vs SM*** | MD | 2.73 | -0.39 to 5.84 | 0.086 | 0.688 | 0.161 |
|  |  | **OS vs SM*** | MD | 2.95 | -0.16 to 6.07 | 0.063 | 0.566 | 0.135 |
|  | **T12** | **PBM vs OS** | MD | -0.32 | -3.43 to 2.79 | 0.841 | 1.000 | 0.950 |
|  |  | **PBM vs SM*** | MD | 5.41 | 2.30 to 8.52 | <0.001 | 0.007 | 0.002 |
|  |  | **OS vs SM*** | MD | 5.73 | 2.61 to 8.84 | <0.001 | 0.004 | 0.002 |
|  | **T24** | **PBM vs OS** | MD | -0.67 | -3.81 to 2.46 | 0.674 | 1.000 | 0.843 |
|  |  | **PBM vs SM*** | MD | 5.33 | 2.18 to 8.49 | <0.001 | 0.009 | 0.002 |
|  |  | **OS vs SM*** | MD | 6.01 | 2.87 to 9.14 | <0.001 | 0.002 | 0.001 |
|  | **T48** | **PBM vs OS** | MD | -1.20 | -4.36 to 1.95 | 0.456 | 1.000 | 0.671 |
|  |  | **PBM vs SM*** | MD | 5.63 | 2.45 to 8.81 | <0.001 | 0.006 | 0.002 |
|  |  | **OS vs SM*** | MD | 6.83 | 3.67 to 9.98 | <0.001 | <0.001 | <0.001 |
| **Masticatory muscle tender points** | **T2** | **PBM vs OS** | RR | 0.99 | 0.66 to 1.49 | 0.959 | 1.000 | 0.959 |
|  |  | **PBM vs SM*** | RR | 0.87 | 0.64 to 1.17 | 0.352 | 1.000 | 0.385 |
|  |  | **OS vs SM*** | RR | 0.88 | 0.66 to 1.16 | 0.360 | 1.000 | 0.385 |
|  | **T4** | **PBM vs OS** | RR | 0.76 | 0.46 to 1.27 | 0.293 | 1.000 | 0.366 |
|  |  | **PBM vs SM*** | RR | 0.49 | 0.33 to 0.73 | <0.001 | 0.004 | 0.001 |
|  |  | **OS vs SM*** | RR | 0.65 | 0.47 to 0.90 | 0.011 | 0.085 | 0.020 |
|  | **T12** | **PBM vs OS** | RR | 0.49 | 0.22 to 1.07 | 0.074 | 0.441 | 0.110 |
|  |  | **PBM vs SM*** | RR | 0.18 | 0.09 to 0.34 | <0.001 | <0.001 | <0.001 |
|  |  | **OS vs SM*** | RR | 0.36 | 0.23 to 0.57 | <0.001 | <0.001 | <0.001 |
|  | **T24** | **PBM vs OS** | RR | 0.45 | 0.13 to 1.53 | 0.202 | 1.000 | 0.276 |
|  |  | **PBM vs SM*** | RR | 0.13 | 0.05 to 0.35 | <0.001 | <0.001 | <0.001 |
|  |  | **OS vs SM*** | RR | 0.28 | 0.14 to 0.55 | <0.001 | 0.003 | <0.001 |
|  | **T48** | **PBM vs OS** | RR | 0.10 | 0.01 to 0.72 | 0.022 | 0.157 | 0.037 |
|  |  | **PBM vs SM*** | RR | 0.04 | 0.01 to 0.23 | <0.001 | 0.005 | 0.001 |
|  |  | **OS vs SM*** | RR | 0.37 | 0.18 to 0.77 | 0.007 | 0.066 | 0.016 |

*Model-based contrasts from mixed-effects models: LMM (MD) for VAS/MPMO and Poisson GLMM (RR) for tender points. P-values are unadjusted and adjusted within each outcome across T2–T48 using Holm and Benjamini–Hochberg FDR. T2–T48 denote follow-up weeks. Comparisons involving SM are exploratory (non-randomized).*

**Table S4. Patient Global Impression of Change (PGIC) at weeks 12, 24, and 48: categorized distribution and raw scores.**

**Panel A. Categorized PGIC re-categorized for analysis in Figure 4**

| **Time point** | **Group** | **n** | **Symptom-free** | **Improved** | **Not improved** |
| --- | --- | --- | --- | --- | --- |
| **Week 12** | **SM** | 22 | 0 (0.0%) | 5 (22.7%) | 17 (77.3%) |
|  | **PBM+SM** | 22 | 1 (4.5%) | 21 (95.5%) | 0 (0.0%) |
|  | **OS+SM** | 22 | 0 (0.0%) | 21 (95.5%) | 1 (4.5%) |
| **Week 24** | **SM** | 20 | 0 (0.0%) | 15 (75.0%) | 5 (25.0%) |
|  | **PBM+SM** | 20 | 12 (60.0%) | 8 (40.0%) | 0 (0.0%) |
|  | **OS+SM** | 22 | 8 (36.4%) | 13 (59.1%) | 1 (4.5%) |
| **Week 48** | **SM** | 19 | 1 (5.3%) | 18 (94.7%) | 0 (0.0%) |
|  | **PBM+SM** | 19 | 14 (73.7%) | 5 (26.3%) | 0 (0.0%) |
|  | **OS+SM** | 21 | 15 (71.4%) | 5 (23.8%) | 1 (4.8%) |

**Panel B. Raw PGIC score counts**

| **Time point** | **Group** | **n** | **Score 2 (n)** | **Score 3 (n)** | **Score 4 (n)** | **Score 5 (n)** | **Score 6 (n)** |
| --- | --- | --- | --- | --- | --- | --- | --- |
| **Week 12** | **SM** | 22 | 1 | 16 | 5 | 0 | 0 |
|  | **PBM+SM** | 22 | 0 | 0 | 10 | 11 | 1 |
|  | **OS+SM** | 22 | 0 | 1 | 7 | 14 | 0 |
| **Week 24** | **SM** | 20 | 1 | 4 | 12 | 3 | 0 |
|  | **PBM+SM** | 20 | 0 | 0 | 5 | 3 | 12 |
|  | **OS+SM** | 22 | 1 | 0 | 1 | 12 | 8 |
| **Week 48** | **SM** | 19 | 0 | 0 | 9 | 9 | 1 |
|  | **PBM+SM** | 19 | 0 | 0 | 1 | 4 | 14 |
|  | **OS+SM** | 21 | 0 | 1 | 0 | 5 | 15 |

*Notes: Percentages are based on participants assessed at each time point (excluding missing data). For analysis, PGIC was categorized as symptom-free (score 6), improved (scores 4–5), and not improved (scores 1–3).*
